# Genetic Diversity and Differentiation at Structurally Varying MHC Haplotypes and Microsatellites in Bottlenecked Populations of Endangered Crested Ibis

**DOI:** 10.3390/cells8040377

**Published:** 2019-04-25

**Authors:** Hong Lan, Tong Zhou, Qiu-Hong Wan, Sheng-Guo Fang

**Affiliations:** 1 MOE Key Laboratory of Biosystems Homeostasis & Protection, State Conservation Centre for Gene Resources of Endangered Wildlife, College of Life Sciences, Zhejiang University, Hangzhou 310058, China; amber_lan@163.com (H.L.); zhoutong2016@zju.edu.cn (T.Z.); 2 Department of Agriculture, Zhejiang Open University, Hangzhou 310012, China

**Keywords:** MHC, genetic drift, haplotype, crested ibis, founder effect, bottleneck, conservation genetics, selection

## Abstract

Investigating adaptive potential and understanding the relative roles of selection and genetic drift in populations of endangered species are essential in conservation. Major histocompatibility complex (MHC) genes characterized by spectacular polymorphism and fitness association have become valuable adaptive markers. Herein we investigate the variation of all MHC class I and II genes across seven populations of an endangered bird, the crested ibis, of which all current individuals are offspring of only two pairs. We inferred seven multilocus haplotypes from linked alleles in the Core Region and revealed structural variation of the class II region that probably evolved through unequal crossing over. Based on the low polymorphism, structural variation, strong linkage, and extensive shared alleles, we applied the MHC haplotypes in population analysis. The genetic variation and population structure at MHC haplotypes are generally concordant with those expected from microsatellites, underlining the predominant role of genetic drift in shaping MHC variation in the bottlenecked populations. Nonetheless, some populations showed elevated differentiation at MHC, probably due to limited gene flow. The seven populations were significantly differentiated into three groups and some groups exhibited genetic monomorphism, which can be attributed to founder effects. We therefore propose various strategies for future conservation and management.

## 1. Introduction

Bottlenecked species are prone to genetic drift and depletion of adaptive variation [1], which may raise the probability of extinction owing to inbreeding depression and reduced adaptability [2]. Elucidating the mechanism influencing adaptive genetic variation and differentiation in endangered species is thus informative for evolutionary processes and critical for conservation biology [3,4]. 

The major histocompatibility complex (MHC), an essential component of the vertebrate immune system, is an ideal fitness-relevant marker [5]. This highly polymorphic genetic region contains multigene family members involved in presenting pathogen-derived peptides to T-cells and triggering an adaptive immune reaction [6]. MHC class I molecules, consisting of an α chain and an associated β2-microglobulin, present intracellular antigens to CD8+ T cells, while MHC class II molecules, consisting of α and β chains encoded by separate MHC genes, present extracellular pathogens to CD4+ T cells [6,7]. 

The remarkable level of polymorphism in MHC is thought to result from sexual selection, maternal–fetal interactions, pathogen-mediated selection (PMS), and demographic processes such as genetic drift [7,8,9,10]. There are three nonexclusive mechanisms for PMS: (1) heterozygotes may be favored because they can recognize a greater range of antigens than homozygotes (heterozygote advantage); (2) rare alleles may have a selective advantage as few pathogens are adapted to them (frequency-dependent selection); (3) spatial/temporal heterogeneity in the pathogen load and variation may maintain MHC diversity at the global scale (fluctuating selection) [11]. Evaluating the relative roles of natural selection and genetic drift in driving the maintenance or loss of MHC diversity is challenging, and discordant results have been reported. Some studies found similar population structures between MHC and neutral loci, and it was proposed that genetic drift may overwhelm selection in shaping MHC variation, especially in small and bottlenecked populations [1,12,13]. Elevated MHC differentiation across populations has also been reported, supporting the dominant role of fluctuating selection [14,15,16]. Moreover, limited gene flow among populations can promote local adaptation to consistent pathogen communities, and this spatial variation in pathogen selection regimes may also create more structure in MHC genes [8,17,18]. 

Previous studies of MHC variation were mainly focused at the allele level. However, particularly in birds and reptiles, alleles from different MHC loci are sometimes highly similar and even identical to each other because of frequent gene duplication, conversion, and recombination [15,19,20,21], and the gene copy numbers may also vary among individuals [21,22,23,24,25]. These issues all imply that genotypes and variation estimated on the basis of alleles alone would be unreliable and potentially biased [21]. Even when the MHC loci are reliably separated, analysis based on valid genotypes alone, without considering the linkage among loci (which is often strong), may also bias the results [21]. For example, different results were obtained on the basis of MHC linkage groups compared to individual alleles in populations of the Egyptian vulture [26]. Similarly, certain infections in badgers were associated with MHC class II–class I haplotypes rather than allele presence [27]. Levels of individual fitness can be differentiated more profoundly using multilocus haplotypes. Thus, population analysis using multilocus MHC haplotype with consideration of the sharing of alleles, the copy number variation (CNV), and the linkage among loci may provide more comprehensive knowledge and credible evaluation of the adaptive variation and species fitness. Moreover, it is essential to examine both MHC class I and II genes, in recognition of their different functions, although only a few studies do this. To the best of our knowledge, no avian population study has adopted the MHC class I–class II multilocus haplotype as an adaptive marker.

**Figure 1:**
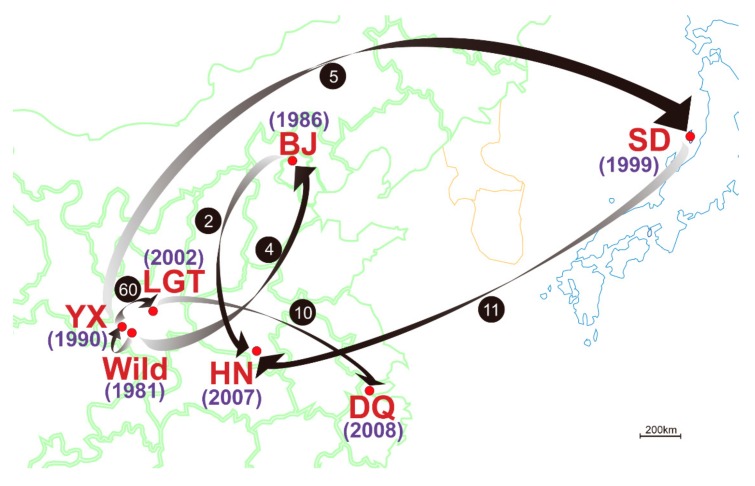
Map of the distribution and establishment of seven crested ibis populations (represented by red dots) in China (green lines) and Japan (blue lines). The SD population is from Sado Island, Japan, while the other populations are all from China. The year that each population was established is given beside the population name. Abbreviations for the six captive populations are: YX, Yangxian; LGT, Louguantai; DQ, Deqing; BJ, Beijing; SD, Sado; HN, Henan. The arrows represent the movement of founders with numbers indicated in the solid black circles.

The crested ibis (*Nipponia nippon*, Pelecaniformes; hereafter referred to as *Nini*), which is listed as “Endangered” according to IUCN criteria [28], was once believed to be extinct in the wild until a tiny population of seven birds (two breeding pairs and three nestlings) was rediscovered in 1981 at Yangxian, China [29]. Since then, the Chinese government have made great efforts to protect this wild population (in-situ conservation) and have also established several captive populations at different sites (ex-situ conservation) (Figure 1, Table 1). Surprisingly, the total number of crested ibis has now increased from seven to over 2000. However, previous studies using mitochondrial DNA and microsatellite markers on small *Nini* populations revealed extremely low levels of polymorphism [30,31]. By analyzing the genomes of 57 historic and 8 modern samples, a recent study revealed ancestral loss of genetic variation and high deleterious mutation owing to genetic drift and inbreeding depression in current populations [32]. There have been a growing number of reports regarding various infections [33,34,35,36] and high mortality [29,33] in this bird, indicating frequent threats from pathogens and undetermined fitness. However, there has not yet been any large-scale genetic assessment on *Nini* populations. It is imperative that such an assessment is completed, preferably by using MHC markers characterized by high polymorphism and fitness relatedness. This could provide insight into the adaptive diversity and differentiation in the recovering populations, thereby better evaluating species fitness and guiding future conservation. Fortunately, the gene architecture of the MHC region has been well illustrated in the crested ibis: ~500 kb MHC region contains five class I genes including one major locus (*UAA*), three minor loci (*UBA*, *UCA1*, and *UCA2*), and one non-classical locus (*UDA*), but the *UAA* locus is located beyond the Class I Region and gathered with the class II genes in the compact Core Region; all these MHC loci were expressed, and different expression levels were found among class I genes [20]. The class IIα and IIβ genes are alternately organized into one to four elementary “αβ” units [20,37,38]. Phylogenetic analysis on the four “αβ” units identified two clusters [20] corresponding to the two reported ancestral avian MHC-IIβ lineages (DAB1 and DAB2) generated by duplication [19]. These results provided a solid foundation for our study. Here, we investigated the variation of all *Nini*-MHC class I and II genes across seven main populations. We further characterized the MHC haplotypes consisting of all class II genes and the major class I gene in the Core Region, and compared the genetic variation and population differentiation at MHC haplotypes with those expected from microsatellites. We aimed to (1) infer the evolutionary history of the structurally varying MHC haplotypes; (2) determine the relative contribution of genetic drift and selective pressures to MHC loci in the bottlenecked populations; (3) interpret the current population structure and provide corresponding conservation and management strategies.

## 2. Materials and Methods

### 2.1. Sampling and DNA Extraction

We collected 307 samples including 141 feather, 130 blood, 25 muscle, and 11 eggshell samples from one wild and six captive populations (Table 1, Table S1). Blood samples were obtained from a wing vein by pricking the skin carefully and collecting a trace of blood on a cotton ball; muscle samples were obtained from dead birds; feather and eggshell samples were collected during molting and breeding times, respectively. All sampling from live birds was approved by the ethics committees of the relevant Breeding Centers, conducted in accordance with the guidelines, and supervised by the technical staff. Genomic DNA was extracted using the standard phenol/chloroform method [39]. 

### 2.2. MHC Genotyping

We analyzed the polymorphic MHC peptide-binding regions (PBR), which are coded by exons 2 and 3 of class I genes and exon 2 of class II genes, using single-strand conformation polymorphism and heteroduplex (SSCP-HD) analysis [40] according to the protocol described by Zhu, et al. [41]. For class I genotyping, given the highly similar introns flanking PBR exons among the five loci [20], we mainly used universal primers and focused on the heteroduplex (HD) banding pattern. An SSCP-HD profile containing the alleles of five class I loci was constructed from the positive bacterial artificial chromosome (BAC) clones stored in our laboratory [20,42], and was verified by profile reconstitution [40]. The profile was supplemented with novel alleles obtained by extracting and cloning new HD bands from genotyping gels (Figure S1a,b). For *UBA* and *UCA2*, which involved a single nucleotide substitution on exon 3, we designed locus-specific primers inside exon 3 and performed precise genotyping focusing on single-strand band patterns (Figure S1c,d). Meanwhile, the exon 3 sequences of UCA1*01 and UCA2*01 were identical. Thus, to avoid the interference from UCA1*01, we also utilized a SNP specific for UCA2*01 on intron 1 to differentiate between UCA2*01/*02 and UCA2*02/*02 through sequencing polymerase chain reaction (PCR) products. For class II genotyping, the eight genes were classified according to their orthologies [20]: For *DAA* and *DAB*, respective locus-specific primers were designed (IIA1e2, IIB1e2) (Figure S1e,f). For *DBA*s (*DBA1*, *DBA2*, and *DBA3*) and *DBB*s (*DBB1*, *DBB2*, and *DBB3*), given the extensive sharing of alleles and identical introns flanking exon 2 among genes of each orthology, orthology-specific primers were designed (IIA2e2, IIB2e2) (Figure S1g,h). Primers for genotyping are illustrated in Table S2. To exclude the possibility of amplification failure of potential alleles, representative genotypes (3 samples for each) were collected for each genotyping and amplified by ≥8 additional valid primers designed for different regions, and the PCR products were sequenced and verified. For *DAB*, an SSCP profile of reference bands was constructed by cloning different genotypes (Figure S1f) [40]. The PCR-SSCP analysis was performed ≥3 times for each primer pair per DNA sample. For cloning and sequencing, we randomly selected ≥5 positive clones per sequence, which were then subjected to Sanger sequencing in both directions by Majorbio (Shanghai, China) using an ABI 3730 automated sequencer. For samples with no PCR product from some primers (putative gene deletion), we extracted DNA twice and designed ≥10 additional sets of primers on both coding (exons 1, 2, 3, and 4) and noncoding (introns 1 and 2) regions to exclude the possibility of poor template quality or PCR failure. All obtained sequences of *Nini*-MHC PBR exons were deposited in GenBank (accession numbers: MK829161–MK829185).

### 2.3. Haplotype Analysis

In this study, we mainly analyzed the *Nini*-MHC haplotypes combining all class II loci and the major class I locus in the Core Region. Linkage disequilibrium (LD) between polymorphic loci (gametic phase unknown) was tested by Arlequin 3.5 [43]. Individual haplotypes were inferred from genotypes both manually and by multiple programs. We first inferred all potential haplotypes from the individuals with 0–1 heterozygous loci (i.e., homozygous at *DAB* and/or *UAA*). For individuals with ≥2 heterozygous loci (i.e., heterozygous at both *DAB* and *UAA*), most haplotypes were deduced using pedigree information provided by reserves (assuming Mendelian inheritance), and the remainder lacking parentage data were phased according to the most frequent haplotypes in their respective populations. Notably, although the crested ibis is strictly monogamous, captive cages are sometimes occupied by >1 breeding pairs (2–3 in most cases), creating the potential for recording false pedigrees. Thus, we verified the pedigrees using microsatellite data, and corrected the paternities of three suspect individuals among candidate parents. Moreover, individual haplotypes were estimated by Haplotype Inference (gametic phase unknown) implemented in Arlequin 3.5 using both Excoffier-Laval-Balding (ELB) and Expectation-Maximization (EM) algorithms (identical results were obtained), and by PHASE 2.1 using a Bayesian statistical method credited with higher accuracy than the EM algorithm [44]. LD coefficients between linked alleles were further computed based on the estimated haplotypes in Arlequin (gametic phase known). Both intra-population and intra-species (combining population data together) analyses were performed.

To determine the genomic structure of the class II region (“*COL11A2*–*BRD2*” fragment) for each haplotype, we randomly selected twelve homozygotes from different populations as follows: four HT02/HT02, three HT03/HT03, one HT07/HT07, two HT04/HT04, and two HT05/HT05 (there were no homozygotes of HT01 and HT06). The BAC clone containing HT01 [20] and an HT01/HT02 heterozygote were chosen as positive controls. We first amplified the full-length sequences of IIβ genes using two sets of primers (3UT1 and 3UT2) (Table S2), and PCR products were cloned and sequenced. Then we amplified the six overlapped segments using the corresponding primers P1–P4, P6, and P7 reported previously [20], and the PCR products of twelve homozygotes were cloned and subjected to primer-walking Sanger sequencing (for both clones and PCR products) by Majorbio (Shanghai, China). Each segment was verified by ≥3 independent rounds of long and accurate PCR (La-PCR). 

### 2.4. Microsatellite Genotyping

After validity assessment of amplification, we first chose 12 reported microsatellite loci for genotyping [30,45]. Each forward primer was labeled with one of the three fluorescent dyes (FAM, HEX, TAMRA), and the triplex PCR products were resolved on the ABI 3730xl DNA analyzer (Applied Biosystems, Foster City, CA, USA). GeneMapper 3.7 (Applied Biosystems) was used to score the genotypes. PCR and genotyping were performed ≥3 times per sample. MICROCHECKER 2.2.1 [46] was used to check for genotyping errors and/or null alleles. All pairs of loci were tested for LD using the exact test (Bonferroni correction) in GENEPOP 4.0 [47]. LD tests were also performed between microsatellite loci and MHC haplotype. One locus was excluded because of its significant LD with another locus in certain populations. Genotypes of 11 loci were used for population analysis (Nn01, Nn03, Nn04, Nn12, Nn16, Nn17, Nn18, Nn21, Nn25, Nn26 [30], and NnNF5 [45]).

### 2.5. Sequence Analysis

Sequences of the *Nini*-IIβ exon 2 obtained above were aligned using the ClustalW algorithm with manual modifications in MEGA 6 [48]. We used two methods to detect historical positive selection on exon 2 of *DAB* and *DBB*. As the exons 2 of *DBB1* and *DBB2* are derived from *DBB3* [20], we integrated the three *DBB* loci as one locus with four alleles (DBB1*01, DBB2*01, DBB2*02, DBB3*01). The selection parameter ω, which estimates the ratio of the nonsynonymous substitution rate (dN) to the synonymous substitution rate (dS), was calculated, and the Z-test with Jukes-Cantor correction was performed in MEGA 6 [48]. Given the limited variation, we input each sequence a number of times equal to its frequency in the primary data. We also used omegaMap 0.5 [49] to detect positive selection under the influence of recombination by simultaneously estimating ω and the recombination rate ρ along the sequence. Analyses of positive selection were performed using a set of objective priors: ω and ρ following an inverse distribution (0.02–500); mutation rate (µ; starting from 0.1), transition/transversion rate (κ; starting from 3.0), and deletion rate (φ; starting from 0.1) following an improper inverse distribution. A variable model was used with a block length of 2 codons. Two independent Markov Chain Monte Carlo (MCMC) chains were performed for 1,000,000 iterations with a thinning interval of 100, and the first 10% of iterations were abandoned as a burn-in. The two chains with high convergence were merged to infer the posterior distribution of ω and ρ. Sites with posterior probability ≥95% were deemed to be under positive selection. R code was used to interpret the test output and generate plots. 

Recombination on the full-length sequences of IIβ genes was detected by the RDP beta 4.16 package [50] using seven methods: RDP, GENECONV, Bootscan, MaxChi, Chimaera, SiScan, and 3Seq. A preliminary scan for recombination was performed with *p* ≤ 0.05 for multiple comparisons. Recombination events identified by at least two methods were then verified and refined by several methods according to the manual, including examination of the plots, matrices, and phylogenetic trees. 

### 2.6. Population Analysis

Haplotype/allele frequencies, and observed (*H_O_*) and expected (*H_E_*) heterozygosities were obtained from Arlequin 3.5. Allelic richness (AR) was calculated using FSTAT 2.9.3 [51], and the correlation between MHC and microsatellites was evaluated using Pearson product-moment correlation in SPSS 20.0. We used Wilcoxon’s test in the program BOTTLENECK [52] to assess evidence of population bottlenecks at microsatellites after Bonferroni correction, with 2000 replicates under the Stepwise Mutation Model.

STRUCTURE 2.3.4 [53] was used to explore population structure using a Bayesian clustering algorithm. Preliminary analysis using standard structure models without information on the sampling location failed to offer a distinct signal of structure, although the pairwise *F*_ST_ showed significant differences among populations. Thus, the admixture model with sampling location as a prior (LOCPRIOR) was used to detect the population structure. The parameter *α* was inferred from the data and *λ* was set to 1. An MCMC chain of 1,000,000 generations was run with an initial burn-in of 100,000 generations. Seven independent replicates of K = 1–7 were performed, and the final number of clusters was determined by simultaneously evaluating posterior probability and the delta K using the online Structure Harvester program [54]. The estimates of r, which evaluates the amount of information carried by the sampling locations, were 0.19 and 0.26 for MHC and microsatellites, respectively, indicating that the sampling locations are informative (near 1 or <1). The plot of the Q matrix was generated by distruct 1.1 [55]. In addition, neighbor-joining (NJ) trees for both markers were constructed to evaluate the genetic distances among populations in Populations 1.2.32 [56] using chord distance. Node supports were evaluated with 2000 bootstrap replicates over individuals.

As expectations for *R*_ST_ may suffer from higher sampling variances under a random mutation process [57], we first performed a size permutation test implemented in SPAGeDi 1.4 [58] to check whether the microsatellite allele sizes carried relevant information on genetic differentiation. As the result of this test was nonsignificant, we then calculated pairwise *F*_ST_ values for both MHC and microsatellites in Arlequin 3.5. Analysis of molecular variance (AMOVA) was conducted on 17 potential groupings with 50,000 permutations in Arlequin 3.5.

*G*’_ST_ was adopted to compare the population structures inferred by MHC and microsatellites because it can control differences in variation among genetic markers [59]. *G*’_ST_ was calculated in R code and 95% confidence intervals (CIs) for pairwise microsatellite *G*’_ST_ values were generated from 1000 bootstrap replications. Then we compared MHC-*G*’_ST_ to the 95% CIs of microsatellite-*G*’_ST_, and the outliers represent population structures that are significantly different from that expected under neutrality. The correlation between MHC-*G*’_ST_ and microsatellite-*G*’_ST_ was tested using Pearson product-moment correlation in SPSS 20.0.

## 3. Results

### 3.1. Characterization of Multilocus MHC Haplotypes 

The MHC class I genotyping showed that *UAA*, *UBA*, and *UCA2* were dimorphic whereas *UCA1* and *UDA* were monomorphic among the crested ibis studied. UBA*02 and UCA2*02 were newly discovered in this study, but differed from UBA*01 and UCA2*01, respectively, by only a single silent nucleotide substitution on exon 3. Thus, only *UAA* was dimorphic at the protein level. The allele frequencies of UAA*01/*02, UBA*01/*02, and UCA2*01/*02 were 0.604/0.396, 0.914/0.086, and 0.500/0.500, respectively. Notably, UBA*02 was only present with UCA2*01, and these two loci showed significant linkage disequilibrium (*p* = 0.000). However, this linkage was not significantly supported between *UAA* and *UBA*–*UCA2* (*p* = 0.083), in accordance with the reported physical distance [20]. Six class I haplotypes were inferred from 213 individuals with 0–1 heterozygous locus (Figure S2).

In the class II exon 2 genotyping, we obtained 1 allele for *DAA* (1 sequence per individual), 1 sequence for *DBA*s (0–1 sequences per individual), 4 alleles for *DAB* (1–2 sequences per individual), and 2 sequences for *DBB*s (one is from *DBB1*, and the other is from *DBB2*/*DBB3*) (0–2 sequences per individual). Three *DBA* genes share the same exon 2; *DBB2* and *DBB3* share the same exon 2. Accordingly, all the class II loci except *DAB* were monomorphic. We also found that *DBA*s and *DBB*s were absent in individuals with DAB*04, whereas DBB1*01 was only present with DAB*01. 

**Figure 2:**
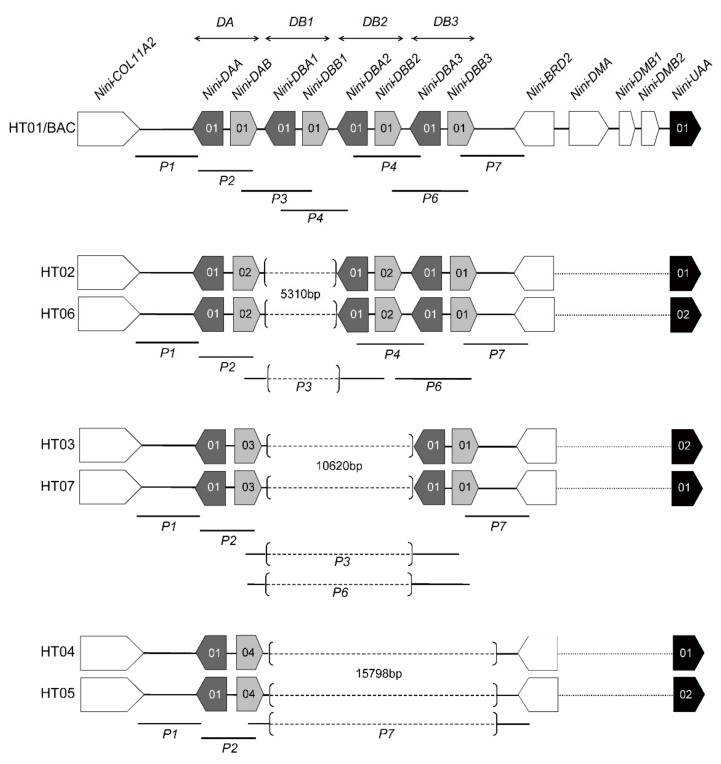
Genomic structures of seven crested ibis MHC haplotypes. A total of seven haplotypes were inferred from genotype data, and their genomic structures of class II region were further determined by La-PCR using representative homozygotes. Dark and light grey boxes indicate the IIα and IIβ genes, respectively, with numbers indicating different alleles on the full-length level. Black boxes indicate the major class I gene (*UAA*) and white boxes indicate other functional genes [20]. Haplotypes are aligned, with bracketed dash lines representing deleted regions (the lengths were denoted aside) in relative haplotypes. Dotted lines between *BRD2* and *UAA* represent the unsequenced region. Locations of the La-PCR fragments amplified by the six reported sets of primers (P1–P4, P6, and P7) [20] are marked below the haplotypes.

The multilocus MHC haplotype of the Core Region consisted of all class II loci and the major class I locus [20]. Among these loci, only *DAB* and *UAA* were polymorphic, and they were in significant linkage disequilibrium (Table S3). Accordingly, we first read all seven haplotypes (HTs01–07) (Figure 2) from 177 homozygotes, and we found strong linkage between certain alleles: DAB*01 was 100% present with UAA*01; DAB*02 was 98.8% present with UAA*01; DAB*03 was 88.1% present with UAA*02; DAB*04 was almost equally present with UAA*01 and *02. This is consistent with the results of LD test between alleles (Table S3). We further phased 89 heterozygotes according to their verified parentages. For the 28 heterozygotes without pedigree information (from Wild, YX and LGT), HT02 and HT03 were favored in the presence of DAB*02 and DAB*03, respectively, considering their higher frequencies against HT06 and HT07 in all populations (e.g., DAB*02/*04–UAA*01/*02: HT02/HT05, DAB*02/*03–UAA*01/*02: HT02/HT03). The manually inferred haplotypes were consistent with the estimates from PHASE and Arlequin (intra-species level) for each individual. When estimating at intra-population level in Arlequin, three individuals (2 from SD and 1 from BJ) showed inconsistent results with the above-inferred haplotypes. However, given their unusually low phase-frequencies (0.517–0.580, overall level: >0.900), these results should be considered invalid and were likely biased by the relative frequencies of HT04 and HT05. We detected nine recombination events between class II genes and *UAA* in parentages: two HT06 were recombined from HT02, three HT07 from HT03, two HT04 from HT05, and two HT05 from HT04. 

In structural analysis of the class II region using homozygotes, the sequencing results of full-length IIβ sequences showed that *DBB2* and *DBB3* were differentiated by their downstream sequences, and two full-length *DBB2* alleles varying in exon 3 were found, in HT01 and HTs02/06 (Table S4); the electrophoresis of six overlapped long-range segments showed various band patterns among haplotypes (Figure S3), and assembly of the six segments showed that IIα and IIβ genes were alternately organized on each haplotype, with four types of haplotype structure (Table S4). Compared to the four “αβ” units (*DA*, *DB1*, *DB2*, and *DB3*) in HT01, there are only three (*DA*, *DB2*, and *DB3*) in HTs02/06, two (*DA* and *DB3*) in HTs03/07, and one (*DA*) in HTs04/05 (Figure 2). Intriguingly, each type can be distinguished by a unique allele of *DAB*. For HTs02/06, HTs03/07, and HTs04/05, haplotypes within each pair differ by alleles of *UAA*. HT01 was the haplotype of the target BAC clone reported previously [20], and the class II regions of HTs02/06, HTs03/07, and HTs04/05 were identical to the three reported *Nini* class II haplotypes HP3, HP2, and HP1, respectively [38]. 

### 3.2. Positive Selection and Recombination on Nini-MHC-Iiβ

**Figure 3:**
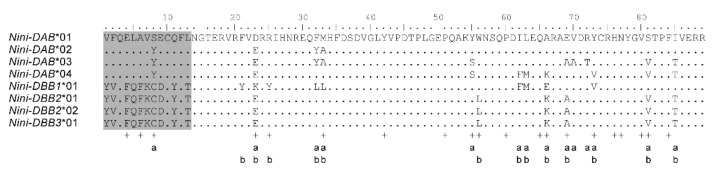
Amino acid alignment of the exon 2 of full-length *Nini*-IIβ sequences. Dots indicate identity to DAB*01. Crosses represent putative antigen binding sites (ABS) inferred from Jardetzky, et al. [60] and Stern, et al. [61]. “a” and “b” denote sites under positive selection for *DAB* and *DBB*s, respectively, as estimated by omegaMap.

The alignment of the *Nini*-IIβ exon 2 amino acid sequences clearly indicated two lineages (*DAB* and *DBB*s) by the first 13 residues (grey regions, Figure 3). Selection analysis on the exon 2 using MEGA revealed a trend towards positive selection on *DAB* (ABS: dN = 0.116, dS = 0.029, *p* = 0.054; non-ABS: dN = 0.020, dS = 0.012, *p* = 0.171) and significant positive selection on *DBB* (ABS: dN = 0.021, dS = 0.000, *p* = 0.004; non-ABS: dN = 0.003, dS = 0.001, *p* = 0.031). All the substitutions on ABS of *DBB* were nonsynonymous. When considering the recombination, omegaMap revealed a mean value of ω = 14.04 per codon and identified fourteen codons under positive selection on the exon 2 of *DAB* (Figure 3 and Figure S4a), of which eight (57%) were in the predicted ABS and the rest were in the immediate vicinity. A higher mean value of ω was obtained (ω = 19.40 per codon) for *DBB* and thirteen positively selected sites were identified (Figure 3 and Figure S4b), of which eight (61.5%) were in the predicted ABS and the rest were adjacent. 

**Figure 4:**
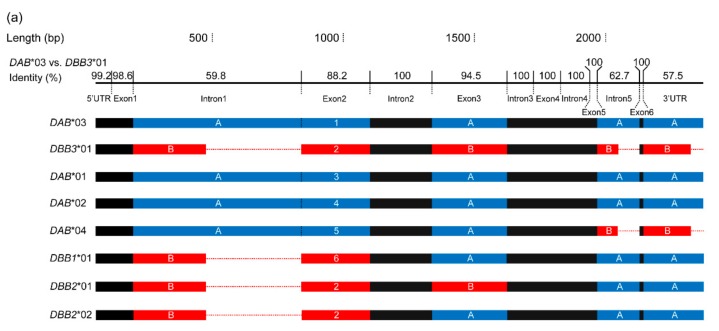

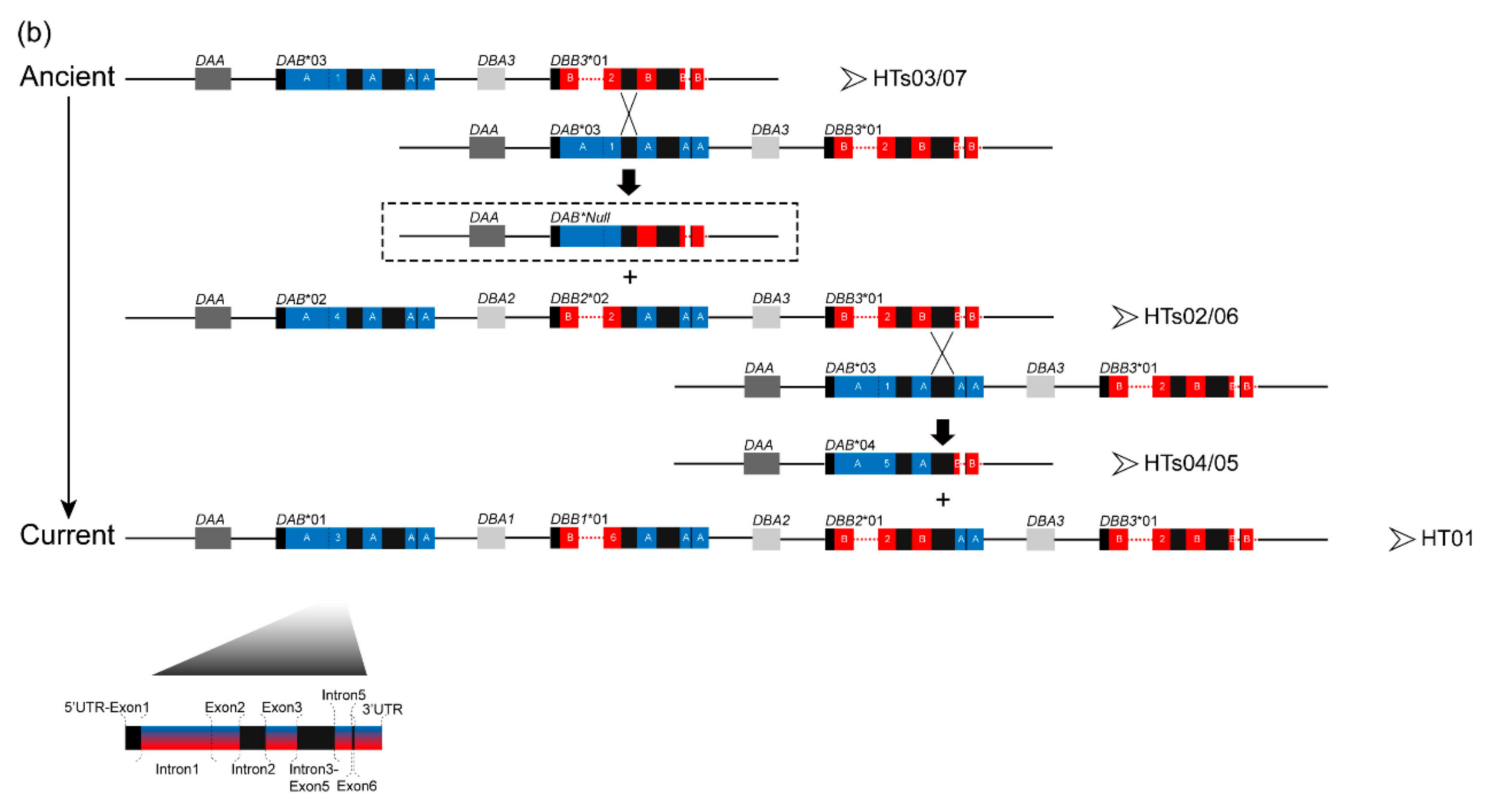
Comparison of the full-length IIβ sequences (**a**) and evolutionary model of the class II regions in seven MHC haplotypes (**b**) in crested ibis. Blue (A) and red (B) boxes respectively indicate “type A” and “type B” regions represented by DAB*03 and DBB3*01. Numbers on exon 2 represent different nucleotide sequences. Black boxes indicate highly similar homologous regions which provide good opportunities for unequal crossing over between two ancestral IIβ genes. Dotted lines indicate alignment gaps. (**a**) Sequence identity (%) for each gene region was calculated in Lasergene software (DNASTAR Inc., Madison, WI) for DAB*03 vs. DBB3*01. (**b**) Dark and light grey boxes represent *DAA* and *DBA*s, respectively. The scale for intragenic regions is shown in the lower left corner. Breakpoints of recombination (as indicated by RDP) are shown by black crosses (intron 2 and intron 3–exon 5). The haplotype framed by the dashed-line was not detected in this study.

RDP analysis on the eight full-length IIβ sequences detected five significant recombination events with the most likely parental sequences of DAB*03 and DBB3*01 (Table S5). This role of recombination in shaping the IIβ sequences was also indicated by their mosaic structures (Figure 4a). Along the whole sequences, most of the nucleotide variation existed in introns 1 and 5, 3′UTR, and exons 2 and 3. All these regions were genetically dimorphic, except exon 2 which was phylogenetically dimorphic (i.e., two lineages). Thus, we designated these dimorphic regions on the parental sequences as “type A” (blue) and “type B” (red) (Figure 4). Accordingly, DAB*01, *02, and *03 contained all “type A” regions, whereas DBB3*01 contained all “type B” regions. The other four IIβ sequences showed mixed patterns. Introns 1, 5, and 3′UTR were far longer in “type A” than in “type B” (intron 1: 660 vs. 285 bp, intron 5: 171 vs. 84 bp, 3′UTR: 244 vs. 199 bp).

### 3.3. Genetic Variation within Populations

Each of the seven populations carried 5 or 6 haplotypes (Table 2). In general, HT02 (33.2%) and HT03 (30.8%) were most common. HT01 (3.9%), HT06 (0.3%), and HT07 (4.3%) were relatively rare and restricted to certain populations. HT01 was only present in group 1, and the only two HT06 detected were products of recombination. Among the four types of haplotypes, HTs02/06 were most prevalent in Wild, LGT, and DQ, while HTs03/07 were most common in BJ, SD, and HN. The majority of individuals in BJ contained HTs03/07. HTs04/05 occurred at higher frequencies in YX and HN than in the other populations.

Of the eleven microsatellite loci, eight were dimorphic and three were trimorphic (Table S6). No population exhibited a significant deficit or excess of heterozygotes for both MHC and microsatellites, except BJ which showed significant heterozygote deficiency for MHC (*p* = 0.015). For both markers, allelic richness (AR) was highest and lowest in Wild and HN, respectively, and decreased progressively in more recently established populations (order of establishment: Wild→ YX→ LGT→ DQ, Wild→ BJ→ HN, Wild→ YX→ SD→ HN). There was a significant correlation (r = 0.805, *p* = 0.029) between the AR values of MHC and microsatellites. The BOTTLENECK analysis supported a significant historical bottleneck in all populations (*p* < 0.005).

### 3.4. Patterns of Population Differentiation

**Figure 5:**
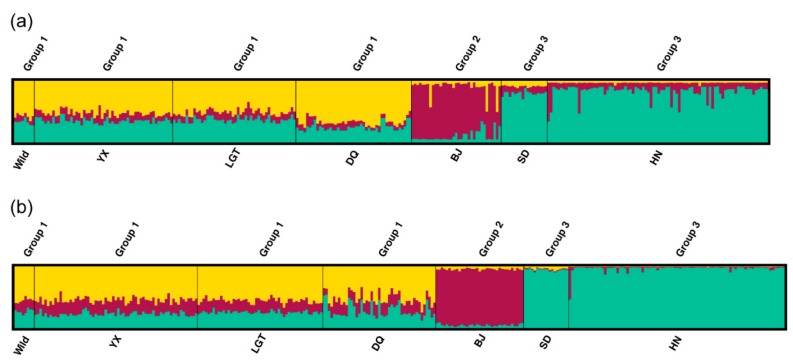
Bayesian clustering analysis (K = 3) using (**a**) MHC haplotypes and (**b**) microsatellite markers. Each individual is represented by a vertical bar, vertically partitioned into segments with lengths proportional to the individual’s estimated membership fraction of each of the three inferred clusters indicated by different colors. The seven populations are separated by black lines and can be divided into three groups based on the proportion of membership of the three clusters.

Clustering analysis using STRUCTURE revealed that both MHC and microsatellite captured the best population genetic structure when K = 3 (Figure S5). According to the proportions of memberships from the three genetic clusters, both analyses allocated individuals from Wild, YX, LGT, and DQ into group 1, individuals from BJ into group 2, and individuals from SD and HN into group 3 (Figure 5). Memberships in group 2 and group 3 were mainly from a single cluster, whereas group 1 contained more even memberships from all three clusters. The NJ trees constructed from MHC and microsatellites shared similar pattern of three major clusters and similar bootstrap supports (Figure S6). The first cluster was “(SD, HN) (BJ)” while the second was “(DQ, LGT) (YX)”. The Wild formed the third cluster as a basal branch, indicating its ancestral position. 

*F*_ST_ values between pairwise populations ranged from −0.036 to 0.199 for MHC and −0.014 to 0.099 for microsatellites (Table S7). AMOVA analysis of 17 potential groupings indicated the highest *F*_CT_ values and most significant *p* values when the grouping was “(Wild, LGT, DQ) (YX) (BJ) (SD, HN)” for MHC and “(Wild, YX, LGT, DQ) (BJ) (SD, HN)” for microsatellites (Table S8). Notably, “(Wild, YX, LGT, DQ) (BJ) (SD, HN)” was the second most likely pattern for MHC, and only slightly less likely than the best grouping (*F*_CT_: 7.29 vs. 7.81, *P* value: 0.010 vs. 0.005) (Table S8). For both markers, most of the variation was found within populations; nonetheless, the level of differentiation among groups was higher than that among populations within groups (Table 3). Similarly, *t*-tests showed that pairwise *F*_ST_ values among groups were significantly higher than those within groups (MHC: *t* = 2.751, d.f. = 19, *p* = 0.040; microsatellite: *t* = 5.064, d.f. = 19, *p* = 0.050) (Table S7). 

**Figure 6:**
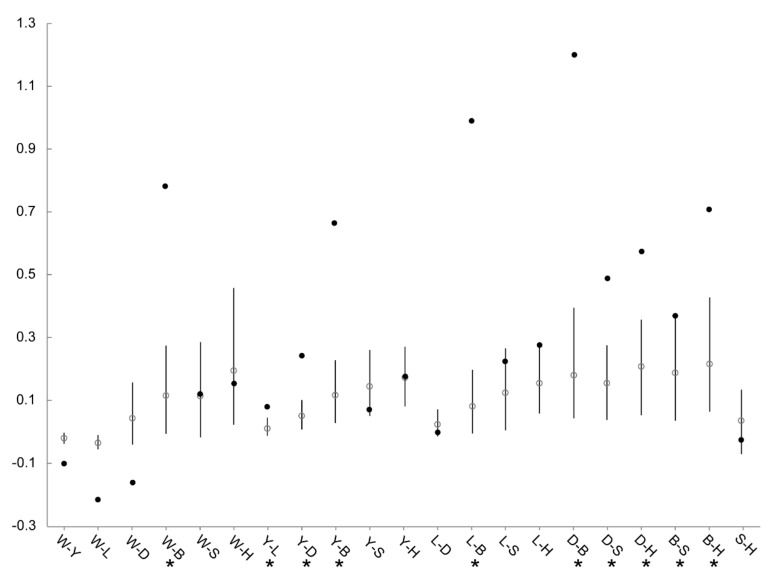
Comparison between pairwise *G*’_ST_ for MHC haplotypes and microsatellite loci among seven populations of the crested ibis. Pairwise *G*’_ST_ with 95% CIs for microsatellite loci is indicated by white circles with corresponding error bars. Black circles indicate pairwise MHC-*G*’_ST_ values. Pairwise populations with significantly higher MHC-*G*’_ST_ than microsatellite-*G*’_ST_ values are marked by asterisks. Populations are W, Wild; Y, YX; L, LGT; D, DQ; B, BJ; S, SD, and H, HN.

The global *G*’_ST_ for MHC haplotypes (0.381) was significantly higher than that for microsatellites (0.126, 99% CI = 0.061–0.212). Meanwhile, 10 of the 21 pairwise MHC-*G*’_ST_ values fell above the 95% CIs of the microsatellites-*G*’_ST_ (Figure 6), and 6 pairs were associated with BJ. Three pairwise MHC-*G*’_ST_ values fell below the 95% CIs of the microsatellites-*G*’_ST_, and these were all pairs from the group 1 suggested by STRUCTURE (W–Y, W–L, and W–D). The mantel test indicated significant correlation between pairwise MHC-*G*’_ST_ and microsatellites-*G*’_ST_ estimates (*r* = 0.660, *P* = 0.001). 

## 4. Discussion

The *Nini*-MHC haplotypes are characterized by different copy numbers of IIαβ units. Evidence for CNVs in MHC class II regions between individuals has been increasingly reported in birds [21,22,23,24,25]. For example, one to two IIαβ units were found across ten MHC class II haplotypes of the Oriental stork [24]; in quail, five MHC-IIβ haplotypes were flexibly organized with 1–7 transcribed IIβ loci [25]. CNV, which emerges faster than other types of mutation, plays a substantial role in generating MHC variation both within and among species [62]. In birds, passerines have strikingly more MHC gene copies than non-passerines. This avian CNV was thought to be correlated with life-history traits (lifespan and migratory behavior) and differences in exposure to pathogens [63]. The evolution of MHC-CNV can be explained by the birth-and-death model that posits that new genes in the multigene family are generated from successive duplication and eventually either maintained or lost [64]. Generally, there are three molecular dynamic mechanisms that may produce CNV: recombination between homologous sequences (unequal crossing over), replication slippage, and retrotransposition [65]. Given the CNV and large-tract intragenic recombination in the MHC class II regions among *Nini* haplotypes, we inferred an evolutionary model using the unequal crossing over mechanism. The previous study on HT01 proposed that *DAA*/*DAB* and *DBA3*/*DBB3* pairs belonging to the two ancestral avian IIαβ lineages might represent the two primitive *Nini*-IIαβ dyads [20]. This speculation is strongly supported by the RDP result, regarding DAB*03 and DBB3*01 on HTs03/07 as the two parental sequences. The other five haplotypes all contain 1–2 recombinant IIβ sequences. Accordingly, we strongly suggest that HTs03/07 represent the ancestral *Nini* haplotypes. Based on the RDP results, we hypothesized that HTs02/06, HTs04/05, and HT01 were successively generated from HTs03/07 through two rounds of unequal crossing over events between *DAB* and *DBB3* (Figure 4b). In addition, the recombination in the “*BRD2*–*DMB2*” region further generated three new haplotypes with either allele of *UAA*. In the studied populations, HT06 and HT07 occupy strikingly lower frequencies than HT02 and HT03, respectively. The only two cases of HT06 (found in LGT and DQ) were recombined from parental HT02. In contrast, most cases of HT07 (found in BJ, SD, and HN) can be traced back to one founder (HT03/HT07) of BJ, born in the wild in 1985. Thus, we suggest an earlier generation and/or higher probability of presence in the initial seven birds for HT07 than HT06. Nevertheless, in view of the higher genetic diversity found in historical samples [32], other unknown MHC haplotypes might have existed prior to the species bottleneck and therefore the haplotype evolution might be more complex than we expected. 

Despite structural diversity of *Nini*-MHC haplotypes, most loci were monomorphic. The class II genes showed higher polymorphism than class I genes, and this is thought to be associated with the stronger selection and gene conversion at class II genes (in non-passerines) [9]. Given the low polymorphism of *Nini*-*UAA*, the crested ibis is predicted to be at high risk for infections by intracellular pathogens, such as bird flu and Newcastle virus [35], and this deserves great attention in conservation and management. The recent genome-wide study on the crested ibis revealed that the contemporary population has only retained a small amount of the ancestral genetic variation [32]. Concordantly, the historical presence of positive selection on *Nini*-IIβ genes (as suggested by MEGA and omegaMap) indicated that the ancestral MHC variation should be high [12,66], and therefore the current low polymorphism is probably caused by genetic drift through species bottleneck. Additionally, the genetic drift still has a predominant role over selection in shaping MHC variation within and across the recovering populations. This is strongly supported by the significant correlations in AR values and pairwise *G*’_ST_ values between MHC and microsatellites, as well as the consistent population structures at MHC and microsatellites in most differentiation analyses. The only exception is that AMOVA supported the differentiation of one more group with MHC than with microsatellites; however, this result was not informative as there was also a pronounced support for an identical grouping between the two markers (Table S8). A previous simulation on bottlenecked populations found that the relative roles of selection and drift in driving MHC diversity depend on the timescales. Initially, selection was not effective in maintaining high polymorphism, but after ~40 post-bottleneck generations, selection overwhelmed the drift and restored variation to pre-bottleneck levels [67]. Nevertheless, high MHC diversity generated by strong selection pressures was reported in bottlenecked fox populations within only ~10–20 generations from 10 individuals [68]. Thus, we call for long-term monitoring of the adaptive diversity in *Nini* populations. With such dramatic loss of MHC variation, average fitness should decline substantially below the pre-bottleneck level, so why did this species successfully thrive within just 38 years? One possibility is that the captive environments under considerable care may serve as a good shelter for this bird, and thus prevent exposure to potential threats such as competitors, predators, and especially the human activities that were considered to be the dominant cause of this species retrogression [32]. Alternatively, the stress from pathogens may be moderate at the present; in this case, the crested ibis is still at risk from catastrophic deterioration caused by novel parasites. Nevertheless, there is little doubt that this bird still maintains a robust reproductive rate. 

The global *G*’_ST_ values suggested elevated differentiation at MHC relative to microsatellites. However, this trend appears to be a by-product of strong MHC differentiation between only a few populations. This claim can be strongly supported by the comparisons of pairwise *G*’_ST_ between two markers (Figure 6). Most outliers with significantly higher MHC-*G*’_ST_ values were associated with BJ. BJ was highly differentiated at MHC from all the other six populations, and obtained extremely high MHC-*G*’_ST_ with the four populations from group 1 (as suggested by STRUCTURE). This might be associated with the unusually high frequency of HT03 (60.0%) and low frequency of HT02 (14.3%) in BJ compared to those in other six populations (Table 2). Given the similar habitat and management across captive populations, the partially higher differentiation at MHC might arise from restricted gene flow translated into different adaptations to local pathogen communities (e.g., reference [18]), rather than fluctuating selection under spatial/temporal heterogeneity in pathogen communities. This is consistent with the demographic history of BJ: as the first captive population, it was established in 1986, and has been almost completely isolated for a long time with restricted in-migrants. This could also explain the significantly lower MHC-*G*’_ST_ than microsatellite-*G*’_ST_ values for three pairs from group 1 (Figure 6). 

The founder effect resulted from improper management is a major cause of genetic drift in conservation programs [69,70,71,72]. Its negative impact is associated with the number and genetic makeup of founders, number of generations (time between founding and sampling), gene flow, reproductive variance, etc [69,71,73]. Ex-situ daughter populations of the same origin may evolve divergently under respective founder effects [71,72]. Thus, it is necessary to investigate and interpret the population structure of the crested ibis after 38 years of conservation practices, thereby evaluating and guiding the captive management [74]. By referring to population histories, we found the current population structure (as suggested by STRUCTURE) could be attributed to inconsistent founder effects across populations. (1) YX (sampled at 7–16 years since establishment) was the second captive population formed with several wild birds, and continuously received injured or sick individuals and abandoned eggs (i.e., gene flow) from the nearby wild population [29]. After 12 years, YX exported 60 individuals as founders to establish LGT (sampled 4 years later). DQ, as the youngest captive population derived from LGT, was sampled within only two generations. Accordingly, these three populations suffered from a relatively weak founder effect and captured richest genetic variation from Wild. Nevertheless, the sample size of Wild is small and sampling time is early (because only 17 wild individuals existed at sampling and unbiased sampling is difficult after its range expansion). Currently, the wild individuals account for nearly half of the total birds. Thus, it is possible that the wild population has already become genetically differentiated from captive populations, especially given that wild individuals are prone to perform mate choice for MHC dissimilarity [7]. (2) In BJ, despite four birds introduced from SD in 2002, most individuals are descendants of a single breeding pair with high reproductive rate but low MHC dissimilarity (HT03/HT03 and HT03/HT07), and BJ had developed for 25 years prior to sampling. Therefore, its founder effect is expected to be extreme, and this was reflected by its isolation with monomorphic genetic resource. Although YX and BJ both originated from the wild around the same time, their different management histories have led to a great distance between BJ (but not YX) and Wild in the NJ trees. (3) Similar to BJ, most individuals in SD (sampled at 12–13 years since establishment) are descendants of two founders from YX. After 8 years of development, 11 descendants were chosen as the main founders of HN. Therefore, the small founder size of group 2 led to its genetic isolation from YX. Notably, the decreasing AR values with establishment of new populations indicate a trend towards continuous genetic drift through the founder effect and inbreeding depression. Accordingly, we call for serious consideration of the anthropogenic founder effect in future conservation management. 

Various markers with different characteristics have been developed for conservation of crested ibis over the past decade. It is important that appropriate markers are chosen for different management issues. The microsatellite markers [30,45] plus the more powerful genome-wide STR markers [75] provide a DNA identification profiling platform, and can be mostly used in demographic management, such as pedigree construction, individual identity and paternity testing. The defensin markers can be applied to improvement of innate immune response among embryos and nestlings [42]. Alternatively, the MHC haplotypes provide a crucial index of individual fitness and population viability. Construction of MHC haplotype profiles, by genotyping *DAB* and *UAA*, was recommended for all reserves. This has already been applied in DQ, as an important reference for conservation strategies, such as artificial pairing, individual exchange, and reintroduction. Moreover, new populations should be established from enough founders with maximum MHC dissimilarity. In order to mitigate the founder effect, we call for appropriate individual exchange between captive populations [70,73]. For example, group 1, which contained the only birds with HT01 and an abundance of HTs02/06, should have more genetic connectivity with groups 2/3 which suffer from limited genetic resources and are rich in HTs03/07. Recently, several reserves have successfully reintroduced captive birds into the wild, providing a giant step forward. However, the low level of adaptive diversity implies a survival risk for these birds with exposure to more diverse pathogen faunas but less artificial care. Similar consideration was also proposed based on the genome-wide diversity loss [32]. Thus, it is crucial to persistently monitor their genetic diversity and population structure using MHC haplotypes and neutral markers. 

Associations between certain MHC haplotypes and infections have been increasingly identified in birds [76,77,78]. In the crested ibis, the structural and genetic variation across haplotypes strongly implies the immunological divergence. HT01, which holds the maximum gene copies including a unique one (*DBB1*), may be the most immunologically competitive haplotype, whereas HTs04/05 may be most susceptible. Nevertheless, the immunity can be confounded by various factors, especially when gene copies function at different levels. A small-scale analysis on the correlation between *Nini*-MHC haplotypes and offspring mortality by our laboratory found a potentially higher mortality in HT03/HT04 heterozygotes [79]. However, the offspring might not have died from infections, so further investigation of *Nini*-MHC haplotype–pathogen relationships should be undertaken for improved conservation. Besides, the MHC haplotypes are ideal markers for studying mate-choice mechanism. 

Here we demonstrated an application of multilocus MHC haplotype in population genetic analysis, which particularly suits to populations with low polymorphism, such as bottlenecked or isolated populations [27,40]. Loci with limited alleles are not informative and can sometimes produce inaccurate analysis results, especially in the presence of CNV and/or shared alleles. In contrast, the MHC haplotype integrating overall variation may provide a more precise assessment of fitness. Individual MHC genes are often in tight linkage that is undesirable in some analyses [21], hence the use of haplotypes recoded from the linked region was suggested as a valid solution (e.g., STRUCTURE [53]). However, we acknowledge that haplotype inference may be hindered for populations with masses of alleles, and given the extensive repeats and high GC-content in the MHC region, the characterization of CNV and shared alleles among haplotypes using La-PCR may be challenging without any prior MHC genomic information. In view of this, the recently-proposed family-assisted inference of the MHC genetic architecture can be an alternative to achieve accurate haplotyping [21]. Notably, whole-genome sequencing has played a promising role in conservation of endangered species including the crested ibis [32,75]. Various estimates can be obtained based on the genome-wide SNPs, including overall genetic diversity, deleterious mutations, phylogenetic relationships, and even population structure [32]. The noncoding SNPs were also considered as suitable neutral markers in MHC variation analysis owing to their similar mutation mechanism [4]. Nevertheless, the assembly of the complicated MHC region remains a challenge for next-generation genome sequencing methods [20,24,37], particularly in non-galliformes, therefore limiting in-depth research on this region.

## Figures and Tables

**Table 1:** Summary of the establishment of seven populations of crested ibis.

Population	Year Established	Founder Size	Sample Size	Sampling Year	Population Size Around (Sampling Year)	Original Population of Founders
Wild	1981	6 ^b^	8	1990–1993	17 (1993)	-
YX	1990	unknown	54/65 *	1997–2006	145 (2004)	Wild
LGT	2002	60	48/50 *	2006	185 (2004)	YX
DQ	2008	10	45	2008–2012	45 (2012)	LGT
BJ ^a^	1986	4	35	2011	35 (2011)	Wild
SD	1999	5	18	2011–2012	120 (2011)	YX
HN	2007	13	86	2012	86 (2012)	11 from SD, 2 from BJ

^a^ Four individuals were introduced from SD into BJ in 2002, and two of these participated in breeding of eight “hybrid” generations. ^b^ The wild population was developed from six of the seven rediscovered birds, as one nestling fell off the nest and was later captively bred. * Numbers before and after the slash represent samples used in MHC and microsatellite genotyping, respectively. Population abbreviations are as in Figure 1.

**Table 2:** Summary of genetic variation and haplotype frequencies across seven populations of crested ibis.

Population			Wild	YX	LGT	DQ	BJ	SD	HN	Total
Sample size (MHC/microsatellite)			8/8	54/65	48/50	45/45	35/35	18/18	86/86	294/307
MHC	Homozygote ^1^		5	34	34	23	27	9	45	177
	Heterozygote ^2^		3	20	14	22	8	9	41	117
	Haplotype frequency (%)	HT01	6.3	7.4	5.2	10.0	0.0	0.0	0.0	3.9
		HT02	43.8	28.7	44.8	51.1	14.3	25.0	28.5	33.2
		HT03	25.0	26.9	21.9	18.9	60.0	36.1	32.0	30.8
		HT04	12.5	19.4	15.6	6.7	2.9	22.2	32.0	18.5
		HT05	12.5	17.6	11.5	12.2	8.6	5.6	1.2	9.0
		HT06	0.0	0.0	1.0	1.1	0.0	0.0	0.0	0.3
		HT07	0.0	0.0	0.0	0.0	14.3	11.1	6.4	4.3
		HTs02/06	43.8	28.7	45.8	52.2	14.3	25.0	28.5	33.5
		HTs03/07	25.0	26.9	21.9	18.9	74.3	47.2	38.4	35.1
		HTs04/05	25.0	37.0	27.1	18.9	11.5	27.8	33.2	27.5
	*H_O_*		0.63	0.78	0.75	0.71	0.54*	0.78	0.81	0.71
	*H_E_*		0.76	0.78	0.72	0.68	0.60	0.76	0.71	0.75
	AR		5.00	4.67	4.59	4.60	4.09	4.61	3.84	4.78
Microsatellite	*H_O_*		0.43	0.50	0.46	0.44	0.49	0.55	0.39	0.45
	*H_E_*		0.49	0.45	0.44	0.43	0.46	0.47	0.37	0.44
	AR		2.27	2.22	2.22	2.14	2.14	2.17	2.13	2.23

^1^ Homozygous at *DAB* and/or *UAA*; ^2^ Heterozygous at both *DAB* and *UAA*; AR, allelic richness; *H_E_*, expected heterozygosity (haplotype level for MHC); *H_O_*, observed heterozygosity (haplotype level for MHC). * significant deficiency of heterozygotes at *P* < 0.05.

**Table 3:** Results from AMOVA of MHC haplotypes and microsatellite loci.

Locus	Grouping	Source of Variation	d.f.	SS	Variance Components	Percentage of Variation	Fixation Index	*P* Value
MHC	(Wild, LGT, DQ) (YX) (BJ) (SD, HN)	Among groups	3	12.800	0.02996	7.81	0.07810	0.00484
		Among populations within groups	3	0.633	−0.00250	−0.65	−0.00706	0.81299
		Within populations	581	206.899	0.35611	92.84	0.07159	0.00000
Microsatellite	(Wild, YX, LGT, DQ) (BJ) (SD, HN)	Among groups	2	49.130	0.10917	5.10	0.05102	0.00897
		Among populations within groups	4	17.424	0.03245	1.52	0.01598	0.00002
		Within populations	607	1212.932	1.99824	93.38	0.06618	0.00000

Significance values are derived from 1023 permutations.

## Supplementary Materials

The following are available online at https://www.mdpi.com/2073-4409/8/4/377/s1. Figure S1. SSCP genotyping patterns. The upper and lower regions show the single-strand and heteroduplex regions, respectively. Arrows indicate the specific bands among genotypes in two class I SSCP-HD profiles. For each photograph, the left part shows representative genotypes, and the right part shows partial genotyping results in population surveys. (a) Exon 2 of class I genes. Numbers 1 to 3 represent *UAA**01/*01, *UAA**02/*02, and *UAA**01/*02. (b) Exon 3 of class I genes. Numbers 1 to 3 represent *UAA**01/*02, *UAA**02/*02, and *UAA**01/*01. (c) Exon 3 of *UBA*. Numbers 1 to 3 represent *UBA**02/*02, *UBA**01/*01, and *UBA**01/*02. (d) Exon 3 of *UCA2*. Number 1 represents *UCA2**01/*01, and number 2 represents *UCA2**01/*02 or *UCA2**02/*02 (see Methods for further differentiation). (e) Exon 2 of *DAA*. (f) Exon 2 of *DAB*. Numbers 1 to 9 represent *DAB**02/*02, *DAB**03/03, *DAB**04/*04, *DAB**01/*02, *DAB**01/*03, *DAB**01/*04, *DAB**02/*03, *DAB**02/*04, and *DAB**03/*04. *DAB**01/*01 was not found. (g) Exon 2 of *DBA*s. (h) Exon 2 of *DBB*s. Number 1 represents the sequence from *DBB2/DBB3*, and number 2 represents the mixed sequences from *DBB1* and *DBB2/DBB3*. The reference band of *DAB**04 was added to each lane to strengthen the bands of *DBB1* with low proportion in mixed PCR products. Figure S2. Six MHC class I haplotypes of crested ibis. Black boxes indicate the five class I genes, with numbers representing alleles of each gene. White boxes indicate other functional genes, and the dotted oval represents the gap between the Core Region and the Class I Region [20]. Figure S3. La-PCR results for six segments of the class II region (P1–P4, P6, and P7) in representative samples. The ladder with reference band sizes (bp) is shown on the left, and the bright reference bands are labelled in red. Figure S4. Spatial variation in the logarithm of the selection parameter (ω) across the exon 2 of (a) *Nini*-*DAB* and (b) *Nini*-*DBB* as estimated with omegaMap. The site-wise mean (solid line) and 95% confidence intervals (grey shaded area) are shown. Values of log(ω) > 0 imply positive selection. The plots of posterior probability and selection parameters overlapped, so only one plot is shown for each analysis. Figure S5. Estimating the true number of clusters with ΔK for (a) MHC haplotypes and (b) microsatellite loci. The uppermost level of structure is the true number of clusters. Figure S6. Neighbor-joining trees of seven crested ibis populations for (a) MHC haplotypes and (b) microsatellites. Bootstrap values were computed over 2000 replicates and are shown as percentages. Table S1. Details of the crested ibis samples analyzed in this study. Table S2. Primers for PBR exon genotyping of *Nini*-MHC genes and amplification of full-length IIβ sequences in haplotype analysis. Table S3. Results of linkage disequilibrium (LD) test from Arlequin. Table S4. Summary of the *Nini*-IIβ sequences amplified by 3UT1 and 3UT2 and long-range segments amplified by P1–P4, P6, and P7 for each haplotype. Table S5. Recombination events between the full-length sequences of *Nini*-IIβ genes, identified using RDP. Table S6. Allele frequencies (%) of microsatellite loci across seven populations of crested ibis. Table S7. Pairwise *F*_ST_ values between populations for MHC (below diagonal) and microsatellites (above diagonal). Table S8. Results of the hierarchical AMOVA for the crested ibis.
