# Supplementary material for: Genetic Diversity and Differentiation at Structurally Varying MHC Haplotypes and Microsatellites in Bottlenecked Populations of Endangered Crested Ibis
Source: Cells. 2019 Apr 25;8(4):377. doi: 10.3390/cells8040377 (PMC6523929; doi:10.3390/cells8040377)
Supplement: Supplementary file 1 [file cells-08-00377-s001.zip › cells-420779-supplementary/suppl-proof/Table S1 Details of the crested ibis samples .pdf]

Table S1 Details of the crested ibis samples analyzed in this study

Population	Location	No. of individuals	Type of samples	Sampling year	Application
Deqing (DQ, N = 45)	Xiazhuhu Crested Ibis Breeding Center, Deqing, China	10	feather	2008	MHC / microsatellite
		15	blood	2010	MHC / microsatellite
		10	blood	2011	MHC / microsatellite
		10	muscle	2012	MHC / microsatellite
Sado (SQ, N = 18)	Sado Crested Ibis Conservation Center, Niigata, Japan	10	blood	2011	MHC / microsatellite
		8	blood	2012	MHC / microsatellite
Beijing (BJ, N = 35)	Beijing Zoo, Beijing, China	12	blood	2011	MHC / microsatellite
		23	feather	2011	MHC / microsatellite
Henan (HN, N = 86)	Dongzhai Crested Ibis Breeding Center, Henan, China	23	feather	2012	MHC / microsatellite
		63	blood	2012	MHC / microsatellite
Wild (N = 8)	Yangxian Crested Ibis Natural Reserve, Shangxi province, China	1	feather	1990	MHC / microsatellite
		6	blood	1993	MHC / microsatellite
		1	feather	1993	MHC / microsatellite
Yangxian (YX, N = 54/65)*	Yangxian Crested Ibis Protection and Breeding Center, Shangxi province, China	5	muscle	1997	MHC / microsatellite
		3	blood	2000	MHC / microsatellite
		10	muscle	2000	MHC / microsatellite
		1	feather	2000	MHC / microsatellite
		11	eggshell	2005	microsatellite
		35	feather	2006	MHC / microsatellite
Louguantai (LGT, N = 48/50)*	Louguantai Crested Ibis Protection and Breeding Center, Shangxi province, China	1	blood	2006	MHC / microsatellite
		2	feather	2006	microsatellite
		47	feather	2006	MHC / microsatellite
Total		294<br>307			MHC<br>microsatellite

*Numbers before and after the slash represent the numbers of individuals used in MHC and microsatellite genotyping, respectively
