# Supplementary material for: Genetic Diversity and Differentiation at Structurally Varying MHC Haplotypes and Microsatellites in Bottlenecked Populations of Endangered Crested Ibis
Source: Cells. 2019 Apr 25;8(4):377. doi: 10.3390/cells8040377 (PMC6523929; doi:10.3390/cells8040377)
Supplement: Supplementary file 1 [file cells-08-00377-s001.zip › cells-420779-supplementary/suppl-proof/Table S2 Primers.pdf]

Table S2 Primers for PBR exon genotyping of *Nini*-MHC genes and amplification of full-length IIβ sequences in haplotype analysis

	Locus	Location	Primer name	Primer sequence (5′→3′)	Size (bp)	*T*a (°C)
MHC class II genotyping	*DAB*	intron 1	IIB1e2-F	CCGGGGTGCTGAGAGCAGC	359	63
		intron 2	IIB1e2-R	GAAATGTTCTGCCACGCACTCAC		
	*DBB*s (*DBB1*/*DBB2*/*DBB3*)	intron 1	IIB2e2-F	TGACCTTCCCGTGTCTGCACGAG	319	57
		intron 2	IIB2e2-R	GAAATGTTCTGCCACGCACTCAC		
	DAA	intron 1	IIA1e2-F	GGGCGGTGGGAAGGGCACTGAC	325	64
		intron 2	IIA1e2-R	CCGTGCCGTGGCCGGGTTGTTA		
	*DBA*s (*DBA1*/*DBA2*/*DBA3*)	intron 1	IIA2e2-F	CCTGGAGGGCGATGAGAA	353	60
		intron 2	IIA2e2-R	CGTGGCCGGGTTGTTAC		
MHC class I genotyping	*UAA*/*UBA*/*UCA1*/*UCA2*/*UDA*	intron 1	Ie2-CF	TGCCCCCCACCTCTGACC	313	61
		intron 2	Ie2-CR	GCCCCCACTCACCCCTGCTCT		
		intron 2	Ie3-CF	TGGTCGCGTTCCAGGGGCTCAC	345	67
		intron 3	Ie3-CR	GGCCGGCACACGCACCCACAG		
	UBA	exon 3	UBAe3-F	CTACGACGGGAGGGACTTCATGGCT	141	61
		exon 3	UBAe3-R	GTGCTCTGCAGGTAATGCTTCCAGT		
	UCA1/*UCA2*	exon 3	UCAe3-F	ACGCTGCAGCGCGTGTAT	195	60

		exon 3	UCAe3-R	ATGCTTCTTCCGCTCAGCTT		
		intron 1	UCAi1-F	TGCCCCCCACCTCTGACC	251	60
		exon 2	UCAi1-R	AGCTGGTGATGGCTCTGAAAGT		
PCR of full-length IIβ sequences	IIβ genes	exon 1	3UT-F*	GTGCTGGGAGCCGACGTGGCTC		
		3'UTR (type1)	3UT1-R	GACAGTGTGTCCCCAGGGCTCTGC	1986	68
		3'UTR (type2)	3UT2-R	CGCGGCGGGGTTGGCAGGGATCTCG	1668	68

*T*a, annealing temperature for each primer pair. *a common forward primer was used to pair with two different reverse primers
