# Supplementary material for: Genetic Diversity and Differentiation at Structurally Varying MHC Haplotypes and Microsatellites in Bottlenecked Populations of Endangered Crested Ibis
Source: Cells. 2019 Apr 25;8(4):377. doi: 10.3390/cells8040377 (PMC6523929; doi:10.3390/cells8040377)
Supplement: Supplementary file 1 [file cells-08-00377-s001.zip › cells-420779-supplementary/suppl-proof/Table S3 Results of linkage disequilibrium (LD) test from Arlequin .pdf]

Table S3 Results of linkage disequilibrium (LD) test from Arlequin

Linked Allele (*DAB*–*UAA*)	Population															
	Wild		YX		LGT		DQ		BJ		SD		HN		Overall	
	D′[a]	LD test[b]	*D′*	LD test	*D′*	LD test	*D′*	LD test	*D′*	LD test	*D′*	LD test	*D′*	LD test	*D′*	LD test
01–01	1.00	χ^2 = 11.470 (*P* = 0.009, 3 d.f.)	1.00*	χ^2 = 65.306 (*P* = 0.000, 3 d.f.)	1.00	χ^2 = 59.369 (P = 0.000, 3 d.f.)	1.00*	χ^2 = 52.419 (*P* = 0.000, 3 d.f.)	/	χ^2 = 17.408 (*P* = 0.000, 2 d.f.)	/	χ^2 = 8.918 (*P* = 0.012, 2 d.f.)	/	χ^2 = 86.545 (*P* = 0.000, 2 d.f.)	1.00*	χ^2 = 259.219 (*P* = 0.000, 3 d.f.)
01–02	-1.00		-1.00		-1.00		-1.00		/		/		/		-1.00	
02–01	1.00*		1.00*		0.93*		0.93*		1.00*		1.00*		1.00*		0.97*	
02–02	-1.00		-1.00		-0.93		-0.93		-1.00		-1.00		-1.00		-0.97	
03–01	-1.00		-1.00		-1.00		-1.00		-0.51		-0.75		-0.94		-0.80	
03–02	1.00*		1.00*		1.00*		1.00*		0.51*		0.75*		0.94*		0.80*	
04–01	-0.20		-0.05		-0.12		-0.48		-0.20		0.52		0.89*		0.18	
04–02	0.20		0.05		0.12		0.48*		0.20		-0.52		-0.89		-0.18	

[a] Standard disequilibrium value ($D' = D/D_{max}$), calculated between linked alleles based on estimated haplotypes (gametic phase known). Significant values are indicated by asterisks ($P < 0.05$). There was no value when a certain *DAB* allele was absent with both *UAA* alleles, and this is indicated by a slash. [b] Results of LD tests performed between *DAB* and *UAA* via a likelihood ratio test (gametic phase known).
