# Supplementary material for: Genetic Diversity and Differentiation at Structurally Varying MHC Haplotypes and Microsatellites in Bottlenecked Populations of Endangered Crested Ibis
Source: Cells. 2019 Apr 25;8(4):377. doi: 10.3390/cells8040377 (PMC6523929; doi:10.3390/cells8040377)
Supplement: Supplementary file 1 [file cells-08-00377-s001.zip › cells-420779-supplementary/suppl-proof/Table S4 Summary of the La-PCR results.pdf]

Table S4 Summary of the *Nini*-IIβ sequences amplified by 3UT1 and 3UT2 and long-range segments amplified by P1–P4, P6, and P7 for each haplotype

HT num.	Gene organization on each fragment								Structure
	3UT1	3UT2	P1	P2	P3	P4	P6	P7	
HT01	DAB*01	DBB3*01	COL11A2–DAA*01	DAA*01–DAB*01	DAB*01–DBA1*01–DBB1*01	DBA1*01–DBB1*01–DBA2*01 /	DBB2*01–DBA3*01–DBB3*01	DBB3*01–BRD2	**COL11A2–DAA*01–DAB*01–DBA1*01–DBB1*01–DBA2*01–DBB2*01–DBA3*01–DBB3*01–BRD2**
	DBB1*01					DBA2*01–DBB2*01–DBA3*01			
	DBB2*01								
HTs02/06	DAB*02	DBB3*01	COL11A2–DAA*01	DAA*01–DAB*02	DAB*02–DBA2*01–DBB2*02	DBA2*01–DBB2*02–DBA3*01	DBB2*02–DBA3*01–DBB3*01	DBB3*01–BRD2	**COL11A2–DAA*01–DAB*02–DBA2*01–DBB2*02–DBA3*01–DBB3*01–BRD2**
	DBB2*02								
HTs03/07	DAB*03	DBB3*01	COL11A2–DAA*01	DAA*01–DAB*03	DAB*03–DBA3*01–DBB3*01	-	DAB*03–DBA3*01–DBB3*01	DBB3*01–BRD2	**COL11A2–DAA*01–DAB*03–DBA3*01–DBB3*01–BRD2**

HTs04/05	-	*DAB**04	*COL11A2*–*DAA**01	*DAA**01–*DAB**04	-	-	-	*DAB**04–*BRD2*	***COL11A2*–*DAA**01–*DAB**04–*BRD2***

- indicates no PCR product (i.e. gene deletion). Gene organizations of the entire "*COL11A2*—*BRD2*" region on seven haplotypes are shown in bold.
