# Supplementary material for: Genetic Diversity and Differentiation at Structurally Varying MHC Haplotypes and Microsatellites in Bottlenecked Populations of Endangered Crested Ibis
Source: Cells. 2019 Apr 25;8(4):377. doi: 10.3390/cells8040377 (PMC6523929; doi:10.3390/cells8040377)
Supplement: Supplementary file 1 [file cells-08-00377-s001.zip › cells-420779-supplementary/suppl-proof/Table S5 Recombination events by RDP .pdf]

Table S5 Recombination events between the full-length sequences of *Nini*-IIβ genes, identified using RDP.

Recombinant Sequences	Beginning breakpoint	Ending breakpoint	Likely Parental Sequences
*DBB1**01	831 (intron 2)	n.d.	*DBB3**01, *DAB**03
	537 (exon 2)	n.d.	*DAB**04, unknown
*DBB2**01	1328 (intron 3)	n.d.	*DBB3**01, *DAB**03
*DBB2**02	831 (intron 2)	n.d.	*DBB3**01, *DAB**03
*DAB**04	1705 (intron 3)	n.d.	*DBB3**01, unknown

n.d. means not determined.
