# Supplementary material for: Genetic Diversity and Differentiation at Structurally Varying MHC Haplotypes and Microsatellites in Bottlenecked Populations of Endangered Crested Ibis
Source: Cells. 2019 Apr 25;8(4):377. doi: 10.3390/cells8040377 (PMC6523929; doi:10.3390/cells8040377)
Supplement: Supplementary file 1 [file cells-08-00377-s001.zip › cells-420779-supplementary/suppl-proof/Table S6 Allele frequencies (%) of microsatellite loci.pdf]

Table S6 Allele frequencies (%) of microsatellite loci across seven populations of crested ibis

Locus	Allele	Population						
		Wild	YX	LGT	DQ	BJ	SD	HN
NN01	199	31.3	39.2	31.0	46.7	30.0	16.7	9.9
	205	25.0	11.5	12.0	5.6	8.6	22.2	16.3
	197	43.8	49.2	57.0	47.8	61.4	61.1	73.8
NN03	297	81.3	86.2	87.0	100.0	55.7	100.0	97.1
	303	18.8	13.8	13.0	0.0	44.3	0.0	2.9
NN04	213	50.0	36.2	40.0	21.1	42.9	52.8	58.1
	215	50.0	63.8	60.0	78.9	57.1	47.2	41.9
NN12	238	37.5	43.8	28.0	23.3	34.3	25.0	59.3
	240	31.3	13.8	36.0	40.0	22.9	33.3	25.6
	242	31.3	42.3	36.0	36.7	42.9	41.7	15.1
NN16	236	31.3	23.1	21.0	17.8	15.7	30.6	14.0
	240	68.8	76.9	79.0	82.2	84.3	69.4	86.0
NN17	286	50.0	35.4	39.0	24.4	35.7	11.1	5.8
	290	50.0	64.6	61.0	75.6	64.3	88.9	94.2
NN18	158	18.8	8.7	7.6	14.4	12.9	52.8	18.0
	172	43.8	51.0	51.1	34.4	42.9	22.2	33.1
	174	37.5	40.4	41.3	51.1	44.3	25.0	48.8
NN21	175	62.5	50.8	45.0	43.3	22.9	41.7	32.6
	185	37.5	49.2	55.0	56.7	77.1	58.3	67.4
NN25	174	31.3	36.2	28.0	25.6	24.3	36.1	33.7
	178	68.8	63.8	72.0	74.4	75.7	63.9	66.3
NN26	159	93.8	83.8	86.0	87.8	64.3	69.4	83.7
	167	6.3	16.2	14.0	12.2	35.7	30.6	16.3
NnNF05	133	25.0	30.0	24.0	26.7	20.0	19.4	22.7
	137	75.0	70.0	76.0	73.3	80.0	80.6	77.3
