# Supplementary material for: Genetic Diversity and Differentiation at Structurally Varying MHC Haplotypes and Microsatellites in Bottlenecked Populations of Endangered Crested Ibis
Source: Cells. 2019 Apr 25;8(4):377. doi: 10.3390/cells8040377 (PMC6523929; doi:10.3390/cells8040377)
Supplement: Supplementary file 1 [file cells-08-00377-s001.zip › cells-420779-supplementary/suppl-proof/Table S7 Pairwise FST values between populations .pdf]

Table S7 Pairwise F_{ST} values between populations for MHC (below diagonal) and microsatellites (above diagonal)

	**Microsatellite**	**Group 1**				**Group 2**	**Group 3**	
MHC	Pop.	Wild	LGT	DQ	YX	BJ	SD	HN
Group 1	Wild		**-0.014**	**0.016**	**-0.009**	0.049*	0.032*	0.090***
	LGT	**-0.036**		**0.008**	**0.006**	0.036***	0.022**	0.066***
	DQ	**-0.027**	**0.000**		**0.023***	0.081***	0.045***	0.096***
Group 2	YX	-0.016	0.013*	0.040**		0.046***	0.033***	0.073***
Group 3	BJ	0.135**	0.164***	0.199***	0.110***		0.060***	0.099***
Group 4	SD	0.016*	0.036*	0.079**	0.009	0.058**		**0.017**
	HN	0.026*	0.046***	0.095***	0.029**	0.118***	**-0.005**	

Statistical significance after sequential Bonferroni correction is indicated by asterisks (*P < 0.05, **P < 0.01, ***P < 0.001). The groupings were in accordance with AMOVA results. F_{ST} values within groups are shown in bold.
