# Supplementary material for: Genetic Diversity and Differentiation at Structurally Varying MHC Haplotypes and Microsatellites in Bottlenecked Populations of Endangered Crested Ibis
Source: Cells. 2019 Apr 25;8(4):377. doi: 10.3390/cells8040377 (PMC6523929; doi:10.3390/cells8040377)
Supplement: Supplementary file 1 [file cells-08-00377-s001.zip › cells-420779-supplementary/suppl-proof/Table S8 Results of the hierarchical AMOVA.pdf]

Table S8 Results of the hierarchical AMOVA for the crested ibis

Grouping tested	MHC			Microsatellite		
	% var	*F*	P	% var	*F*	P
**Group1**						
**(Wild, YX, LGT, DQ) (BJ) (SD, HN)**						
Among groups [F_{CT}]	7.29	0.073	0.010	5.10	0.051	0.009
Among populations within groups [F_{SC}]	0.87	0.009	0.075	1.52	0.016	0.000
Among individuals within populations [F_{ST}]	91.84	0.082	0.000	93.38	0.066	0.000
**Group2**						
**(Wild, LGT, DQ) (YX) (BJ) (SD, HN)**						
Among groups [F_{CT}]	7.81	0.078	0.005	3.98	0.040	0.029
Among populations within groups [F_{SC}]	-0.65	-0.007	0.813	1.67	0.017	0.000
Among individuals within populations [F_{ST}]	92.84	0.072	0.000	94.36	0.056	0.000
**Group3**						
**(Wild) (YX) (LGT, DQ) (BJ) (SD, HN)**						
Among groups [F_{CT}]	7.13	0.071	0.040	3.78	0.038	0.105
Among populations within groups [F_{SC}]	-0.19	-0.002	0.529	1.77	0.018	0.000
Among individuals within populations [F_{ST}]	93.06	0.069	0.000	94.45	0.056	0.000
**Group4**						
**(Wild, YX) (LGT, DQ) (BJ) (SD, HN)**						
Among groups [F_{CT}]	7.22	0.072	0.019	4.25	0.043	0.010
Among populations within groups [F_{SC}]	-0.37	-0.004	0.680	1.40	0.015	0.001
Among individuals within populations [F_{ST}]	92.95	0.071	0.000	94.35	0.057	0.000
**Group5**						
**(Wild, YX) (LGT, DQ) (BJ, SD, HN)**						
Among groups [F_{CT}]	2.92	0.029	0.120	1.15	0.011	0.227
Among populations within groups [F_{SC}]	3.92	0.040	0.000	4.27	0.043	0.000
Among individuals within populations [F_{ST}]	93.16	0.068	0.000	94.58	0.054	0.000
**Group6**						
**(Wild, YX) (LGT, DQ) (BJ, HN) (SD)**						
Among groups [F_{CT}]	1.22	0.012	0.374	0.54	0.005	0.431
Among populations within groups [F_{SC}]	5.20	0.053	0.000	4.74	0.048	0.000

Among individuals within populations [F_{ST}]	93.59	0.064	0.000	94.73	0.053	0.000
**Group7**						
**(Wild, YX, SD) (LGT, DQ) (BJ) (HN)**						
Among groups [F_{CT}]	6.72	0.067	0.026	3.74	0.037	0.048
Among populations within groups [F_{SC}]	0.18	0.002	0.396	1.83	0.019	0.000
Among individuals within populations [F_{ST}]	93.10	0.069	0.000	94.43	0.056	0.000
**Group8**						
**(Wild, YX, LGT, DQ) (BJ, SD, HN)**						
Among groups [F_{CT}]	3.94	0.039	0.083	2.63	0.026	0.057
Among populations within groups [F_{SC}]	3.72	0.039	0.000	3.53	0.036	0.000
Among individuals within populations [F_{ST}]	92.34	0.077	0.000	93.83	0.062	0.000
**Group9**						
**(Wild, YX, LGT, DQ) (BJ, HN) (SD)**						
Among groups [F_{CT}]	2.35	0.227	0.024	2.12	0.021	0.153
Among populations within groups [F_{SC}]	4.59	0.000	0.047	3.74	0.038	0.000
Among individuals within populations [F_{ST}]	93.06	0.000	0.069	94.14	0.059	0.000
**Group10**						
**(Wild, YX, BJ) (LGT, DQ) (HN, SD)**						
Among groups [F_{CT}]	2.93	0.029	0.115	2.85	0.028	0.057
Among populations within groups [F_{SC}]	3.80	0.039	0.000	2.83	0.029	0.000
Among individuals within populations [F_{ST}]	93.28	0.067	0.000	94.32	0.057	0.000
**Group11**						
**(Wild, YX, BJ) (LGT, DQ, SD) (HN)**						
Among groups [F_{CT}]	0.81	0.008	0.312	2.54	0.025	0.086
Among populations within groups [F_{SC}]	5.56	0.056	0.000	3.10	0.032	0.000
Among individuals within populations [F_{ST}]	93.63	0.064	0.000	94.36	0.056	0.000
**Group12**						
**(Wild) (YX, LGT, DQ) (BJ) (SD, HN)**						
Among groups [F_{CT}]	6.44	0.064	0.030	4.72	0.047	0.010
Among populations within groups [F_{SC}]	1.29	0.013	0.024	1.65	0.047	0.000
Among individuals within populations [F_{ST}]	92.27	0.077	0.000	93.64	0.064	0.000
**Group13**						

**(Wild) (YX, LGT, DQ) (BJ, SD, HN)**						
Among groups [F_{CT}]	3.02	0.030	0.169	2.18	0.022	0.131
Among populations within groups [F_{SC}]	4.21	0.043	0.000	3.75	0.038	0.000
Among individuals within populations [F_{ST}]	92.77	0.072	0.000	94.07	0.059	0.000
**Group14**						
**(Wild) (YX, LGT, DQ, BJ) (SD, HN)**						
Among groups [F_{CT}]	-0.25	-0.002	0.456	3.59	0.036	0.047
Among populations within groups [F_{SC}]	6.39	0.064	0.000	2.99	0.031	0.000
Among individuals within populations [F_{ST}]	93.86	0.061	0.000	93.42	0.066	0.000
**Group15**						
**(Wild, YX, LGT, DQ, BJ) (SD, HN)**						
Among groups [F_{CT}]	-0.73	0.007	0.295	4.09	0.041	0.046
Among populations within groups [F_{SC}]	5.80	0.058	0.000	2.84	0.030	0.000
Among individuals within populations [F_{ST}]	93.47	0.065	0.000	93.07	0.069	0.000
**Group16**						
**(Wild) (YX, LGT, DQ, SD) (BJ, HN)**						
Among groups [F_{CT}]	1.56	0.016	0.298	1.78	0.018	0.189
Among populations within groups [F_{SC}]	5.20	0.053	0.000	4.05	0.041	0.000
Among individuals within populations [F_{ST}]	93.23	0.068	0.000	94.17	0.058	0.000
**Group17**						
**(Wild) (YX, SD) (LGT, DQ) (BJ, HN)**						
Among groups [F_{CT}]	1.43	0.014	0.314	0.10	0.001	0.534
Among populations within groups [F_{SC}]	5.03	0.051	0.000	5.11	0.051	0.000
Among individuals within populations [F_{ST}]	93.54	0.065	0.000	94.79	0.052	0.000

Different groupings were tested to identify those which explain a greater percentage of variation (% var) among groups (F_{CT}) than within groups (F_{SC}). F_{CT} is the correlation of random haplotypes/alleles within a group of populations relative to that of random pairs of haplotypes/alleles drawn from the whole population. F_{SC} is the correlation of the molecular diversity of random haplotypes/alleles within populations relative to that of random pairs of haplotypes/alleles drawn from the region. F_{ST} is the correlation of random haplotypes/alleles within populations relative to that of random pairs of haplotypes/alleles drawn from the populations. For MHC and microsatellite, group 2 and group 1 (highlighted) respectively show the highest F_{CT} values with the most significant P values.
