# Supplementary figures and images for: Genetic Diversity and Differentiation at Structurally Varying MHC Haplotypes and Microsatellites in Bottlenecked Populations of Endangered Crested Ibis
Source: Cells. 2019 Apr 25;8(4):377. doi: 10.3390/cells8040377 (PMC6523929; doi:10.3390/cells8040377)

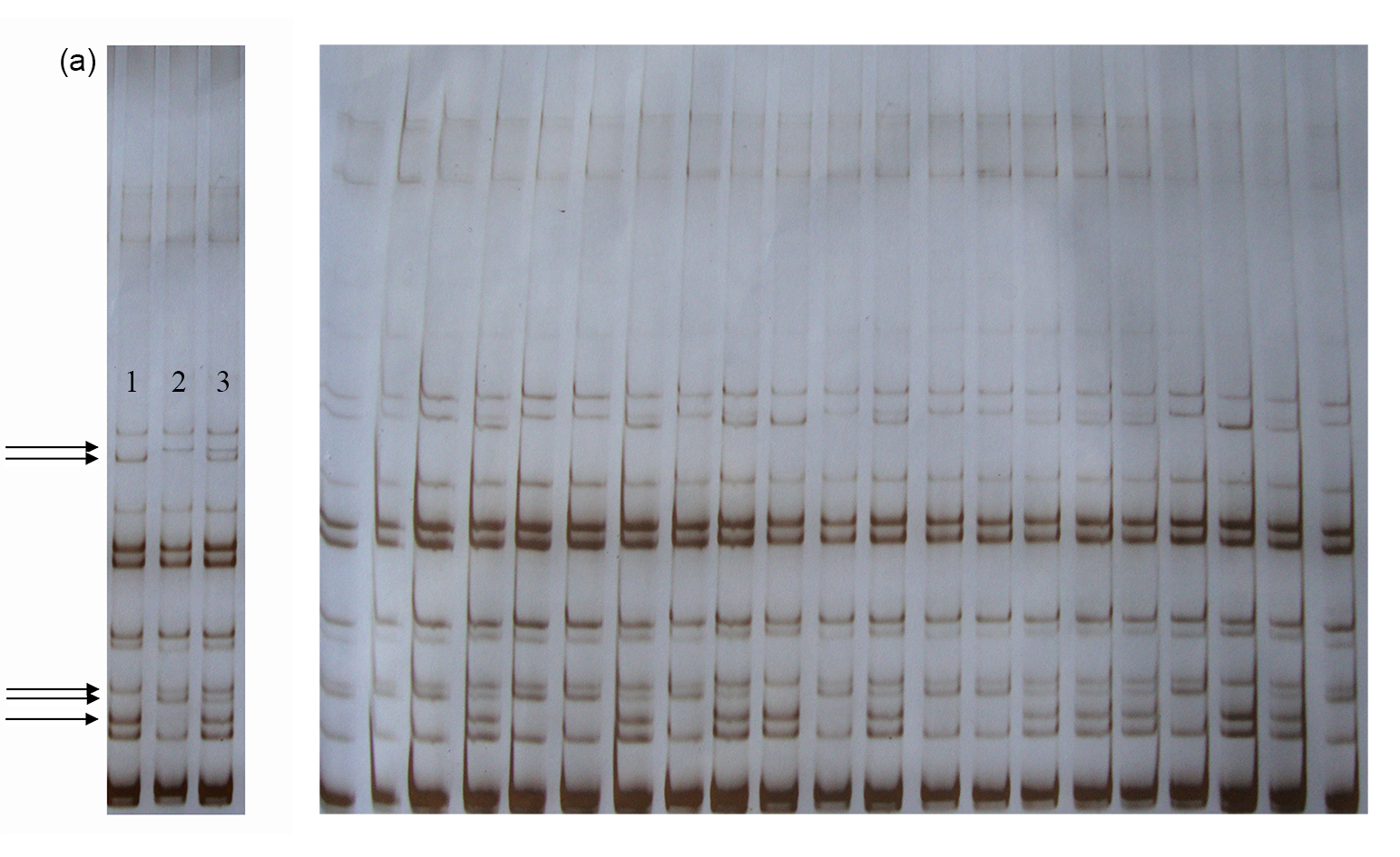

Supplement: Supplementary file 1 [file cells-08-00377-s001.zip › cells-420779-supplementary/suppl-proof/Figure S1a SSCP I-E2.tif]

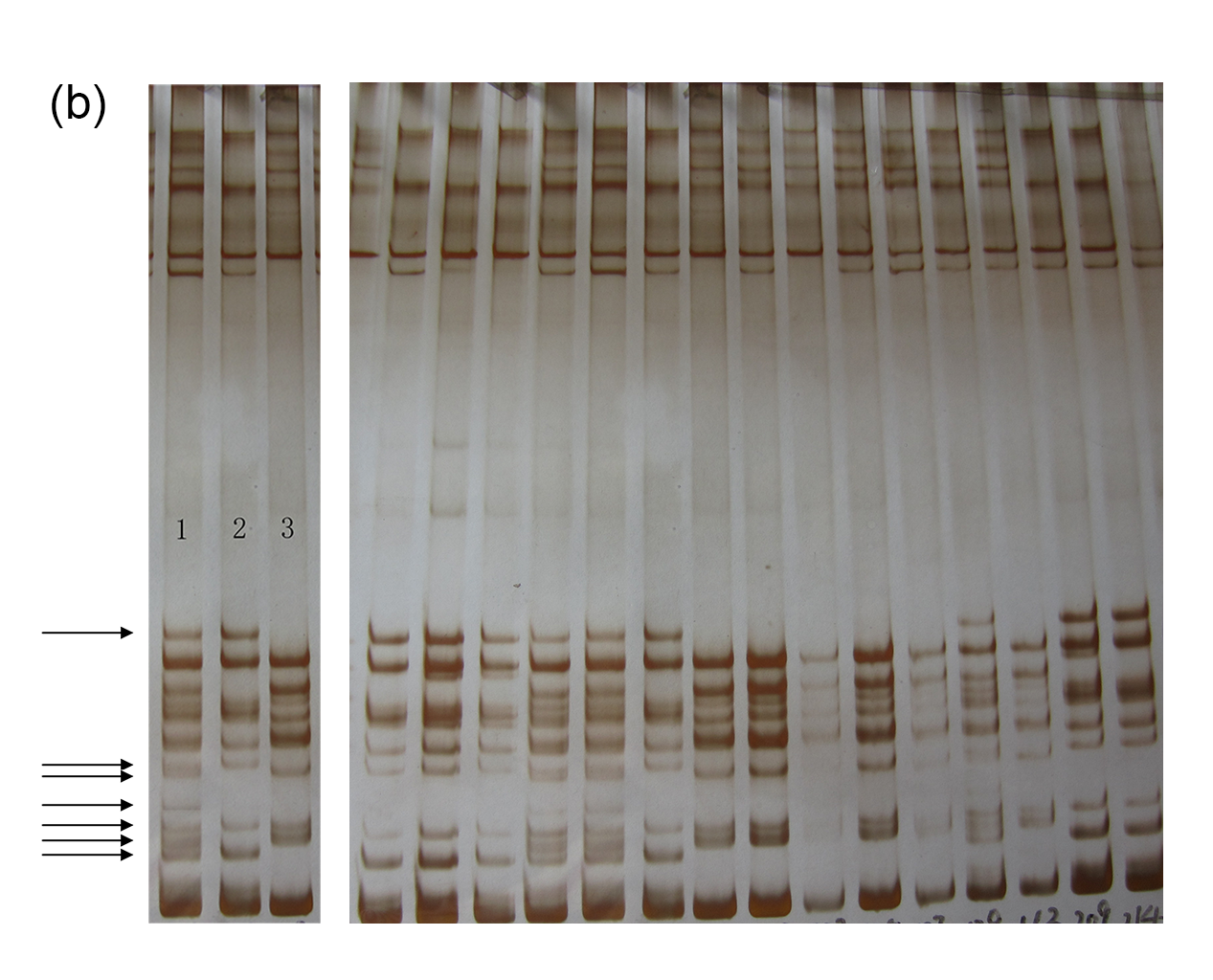

Supplement: Supplementary file 1 [file cells-08-00377-s001.zip › cells-420779-supplementary/suppl-proof/Figure S1b SSCP I-E3.tif]

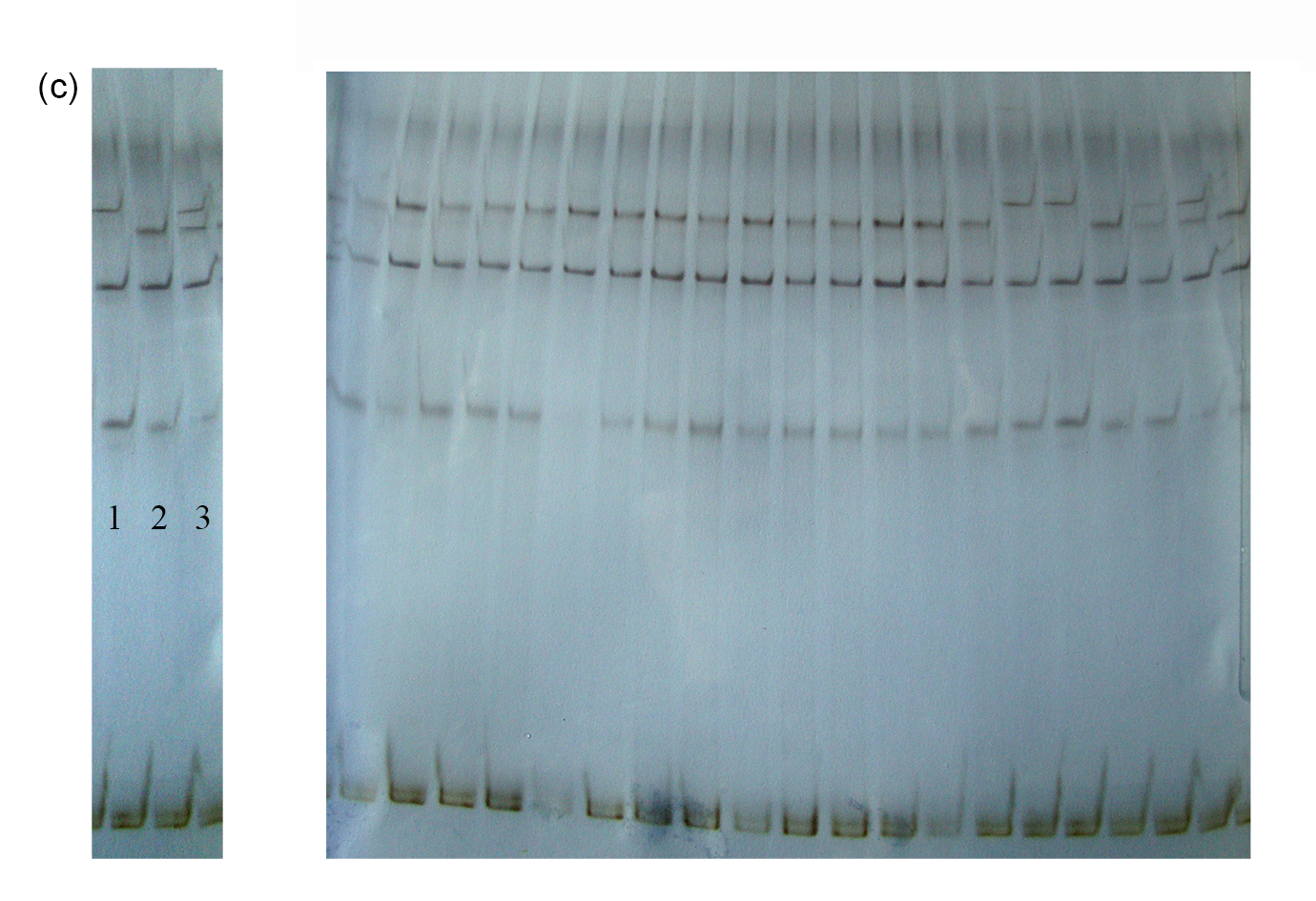

Supplement: Supplementary file 1 [file cells-08-00377-s001.zip › cells-420779-supplementary/suppl-proof/Figure S1c SSCP I-UBA.tif]

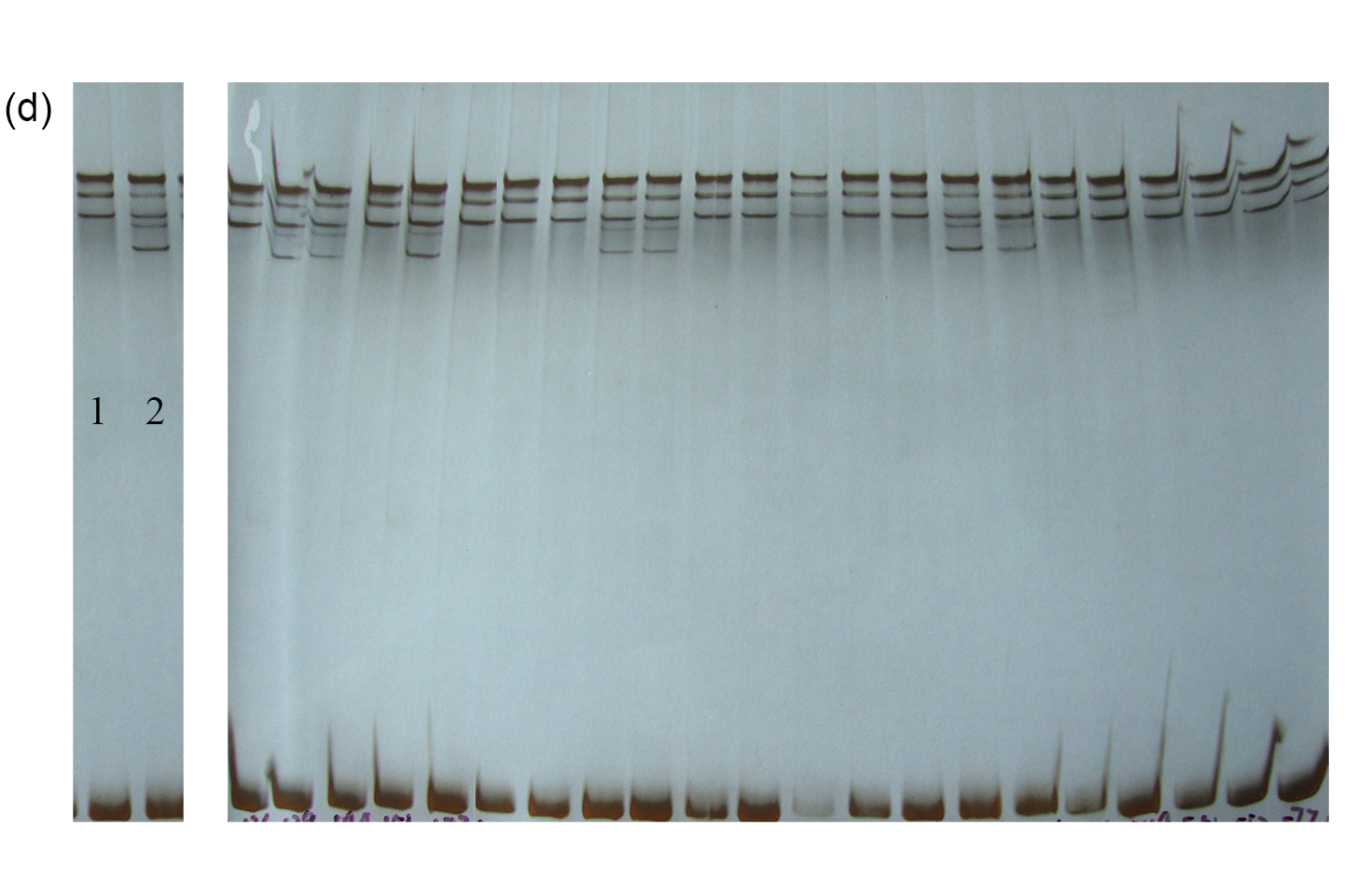

Supplement: Supplementary file 1 [file cells-08-00377-s001.zip › cells-420779-supplementary/suppl-proof/Figure S1d SSCP I-UCA2.tif]

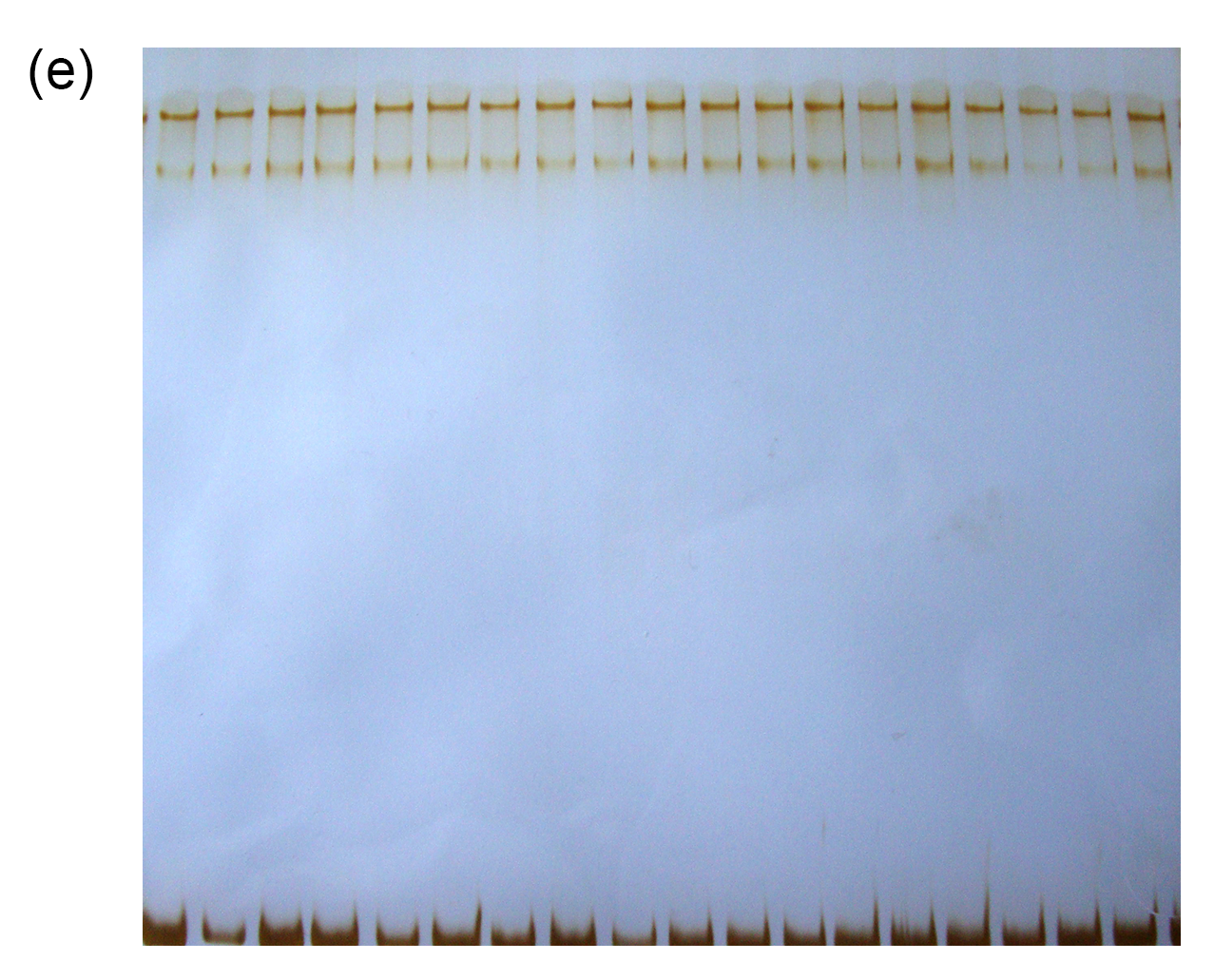

Supplement: Supplementary file 1 [file cells-08-00377-s001.zip › cells-420779-supplementary/suppl-proof/Figure S1e SSCP II-DAA.tif]

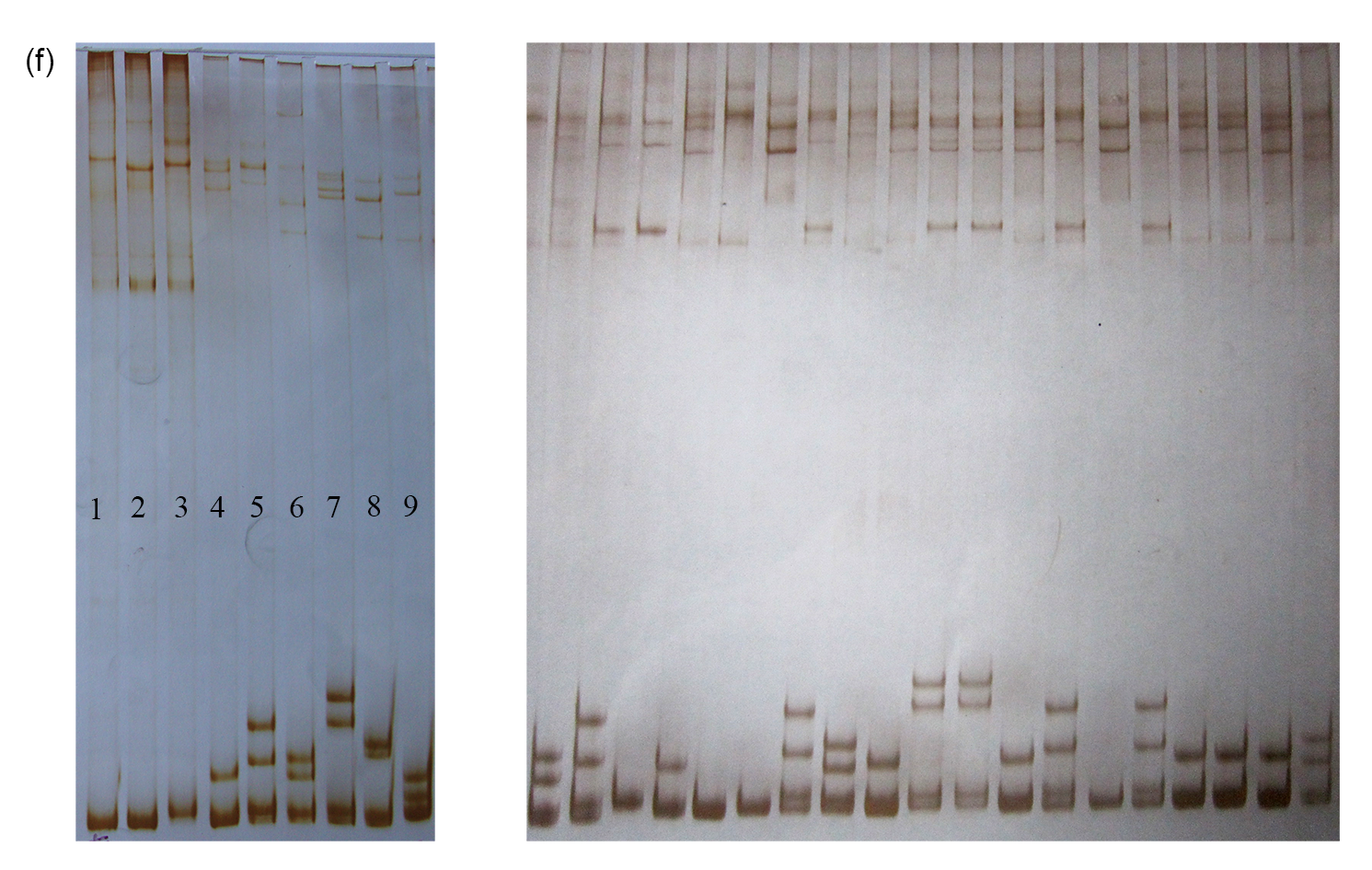

Supplement: Supplementary file 1 [file cells-08-00377-s001.zip › cells-420779-supplementary/suppl-proof/Figure S1f SSCP II-DAB.tif]

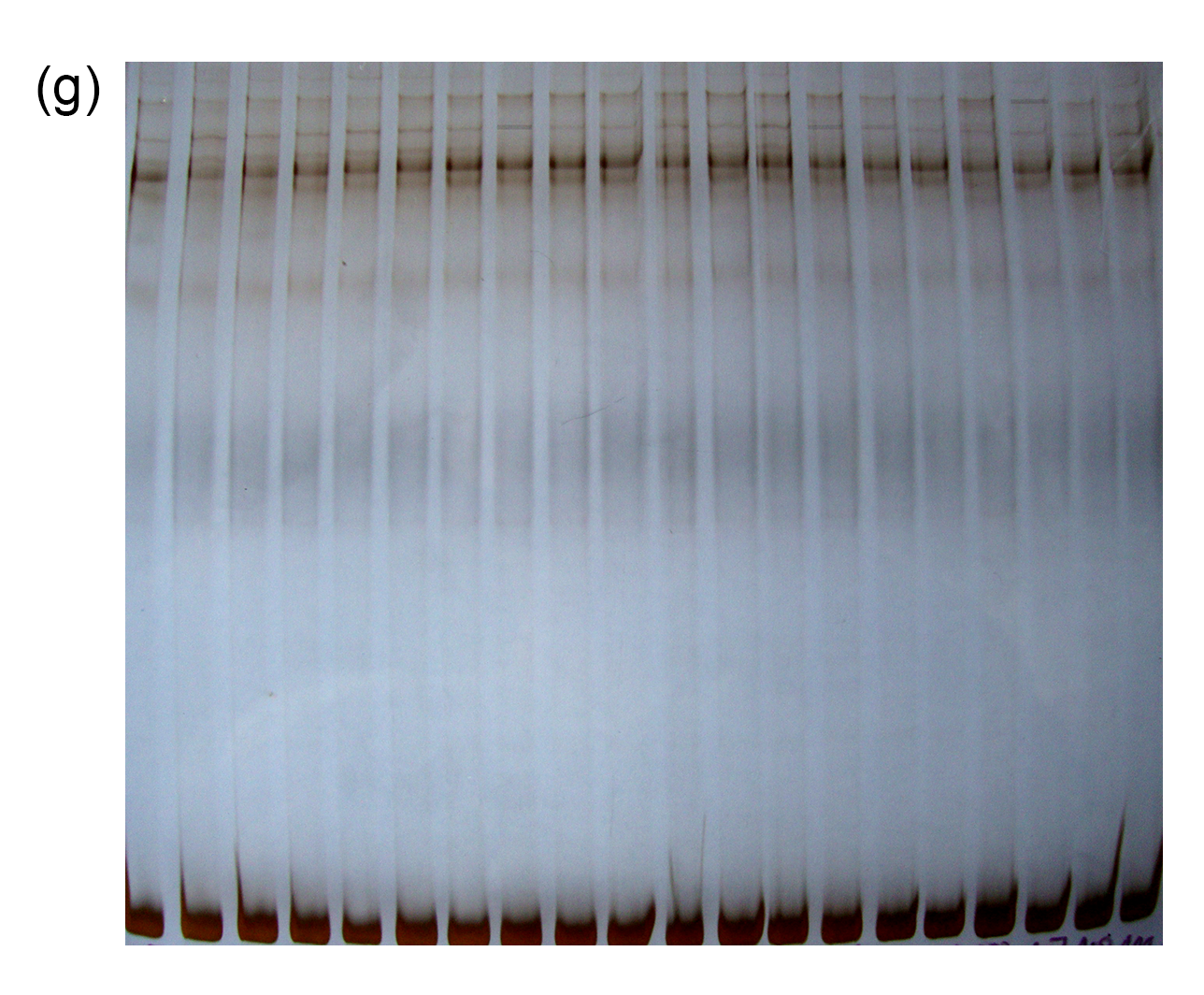

Supplement: Supplementary file 1 [file cells-08-00377-s001.zip › cells-420779-supplementary/suppl-proof/Figure S1g SSCP II-DBAs.tif]

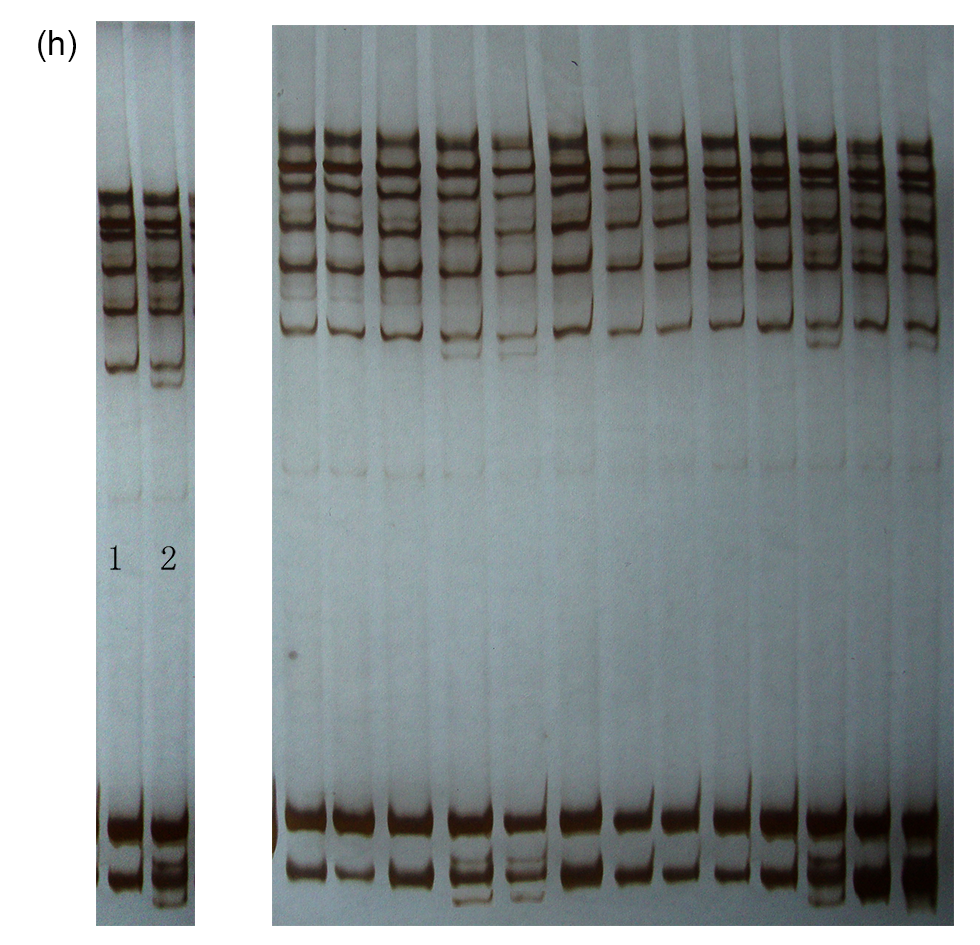

Supplement: Supplementary file 1 [file cells-08-00377-s001.zip › cells-420779-supplementary/suppl-proof/Figure S1h SSCP II-DBBs.tif]

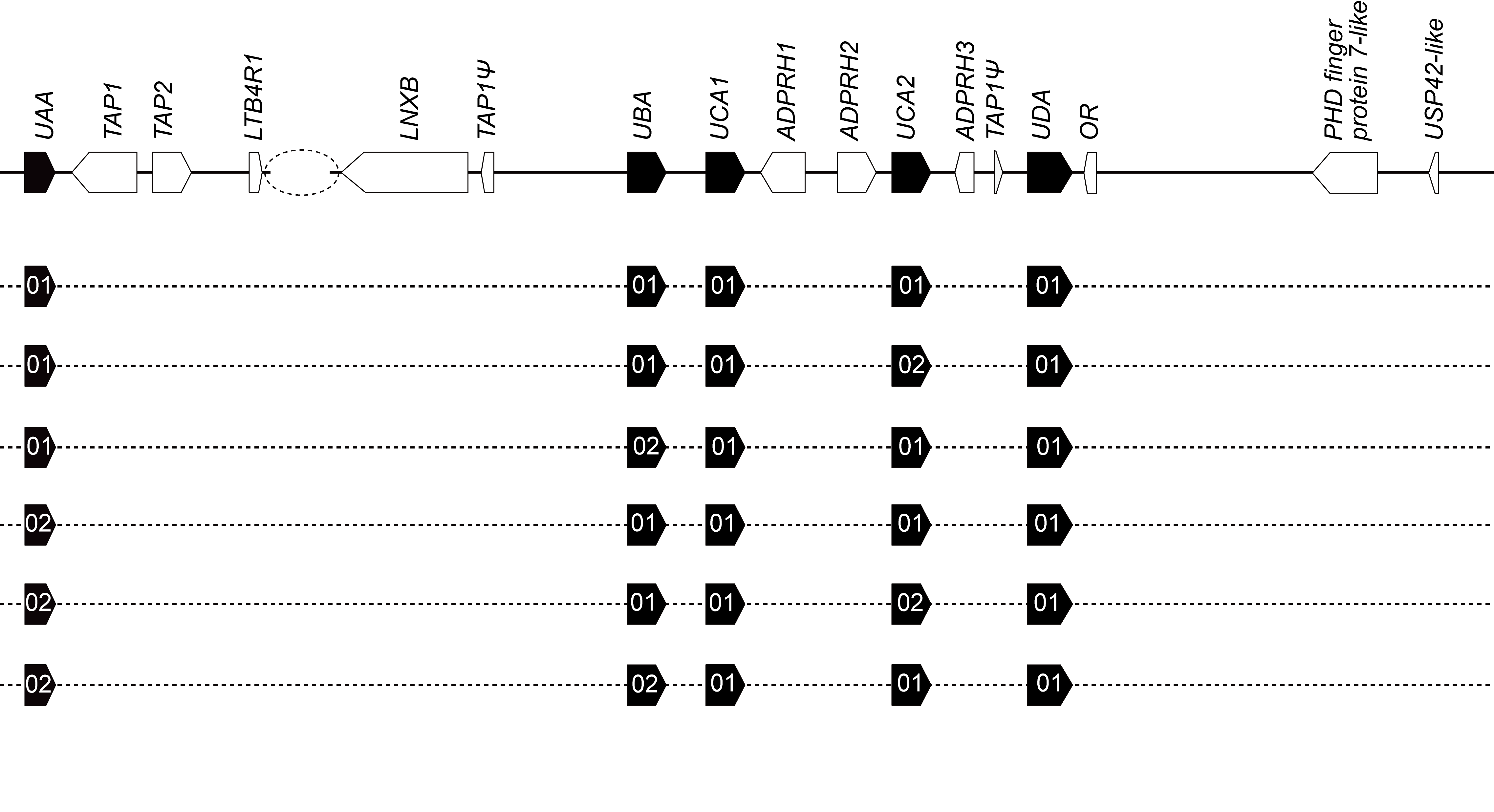

Supplement: Supplementary file 1 [file cells-08-00377-s001.zip › cells-420779-supplementary/suppl-proof/Figure S2 Class I haplotype.tif]

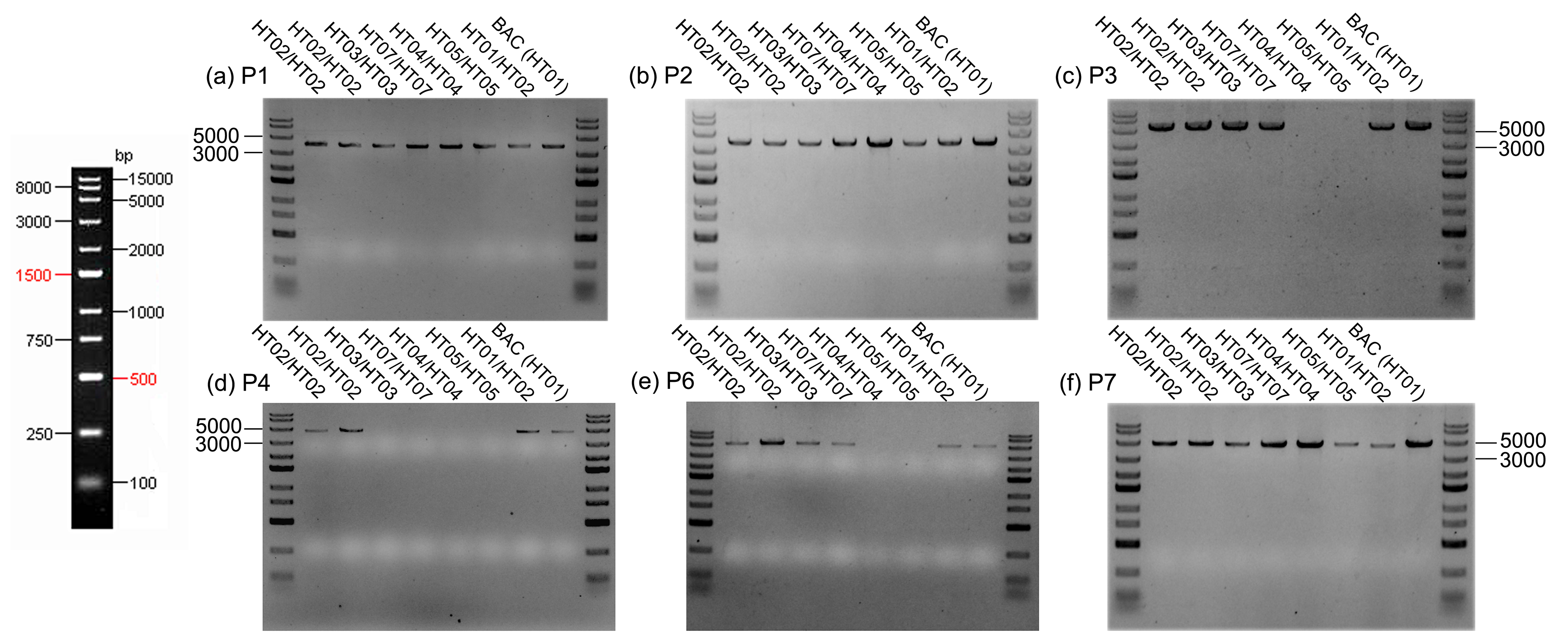

Supplement: Supplementary file 1 [file cells-08-00377-s001.zip › cells-420779-supplementary/suppl-proof/Figure S3 La-PCR electrophoresis results.tif]

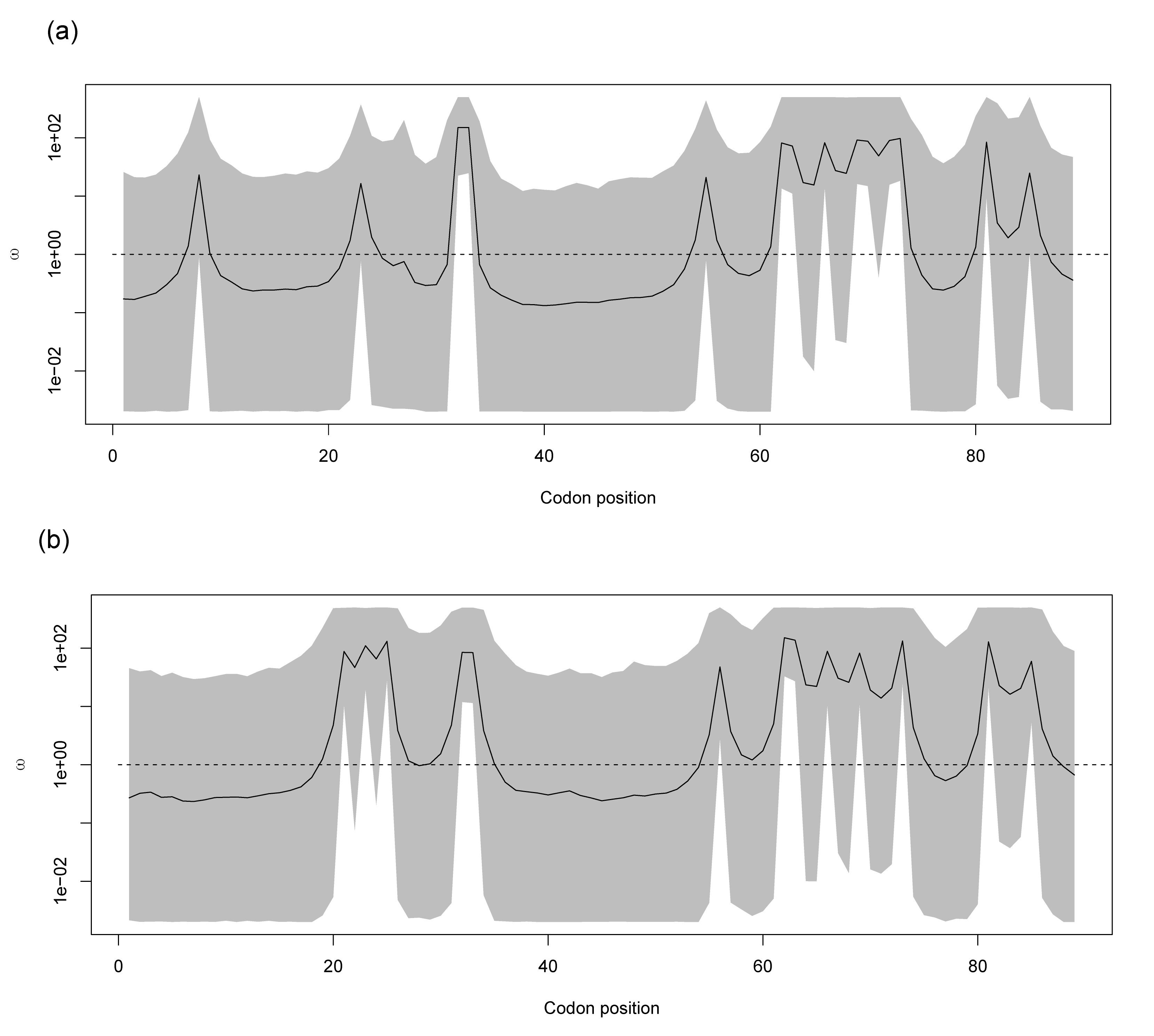

Supplement: Supplementary file 1 [file cells-08-00377-s001.zip › cells-420779-supplementary/suppl-proof/Figure S4 omegaMap Results.tif]

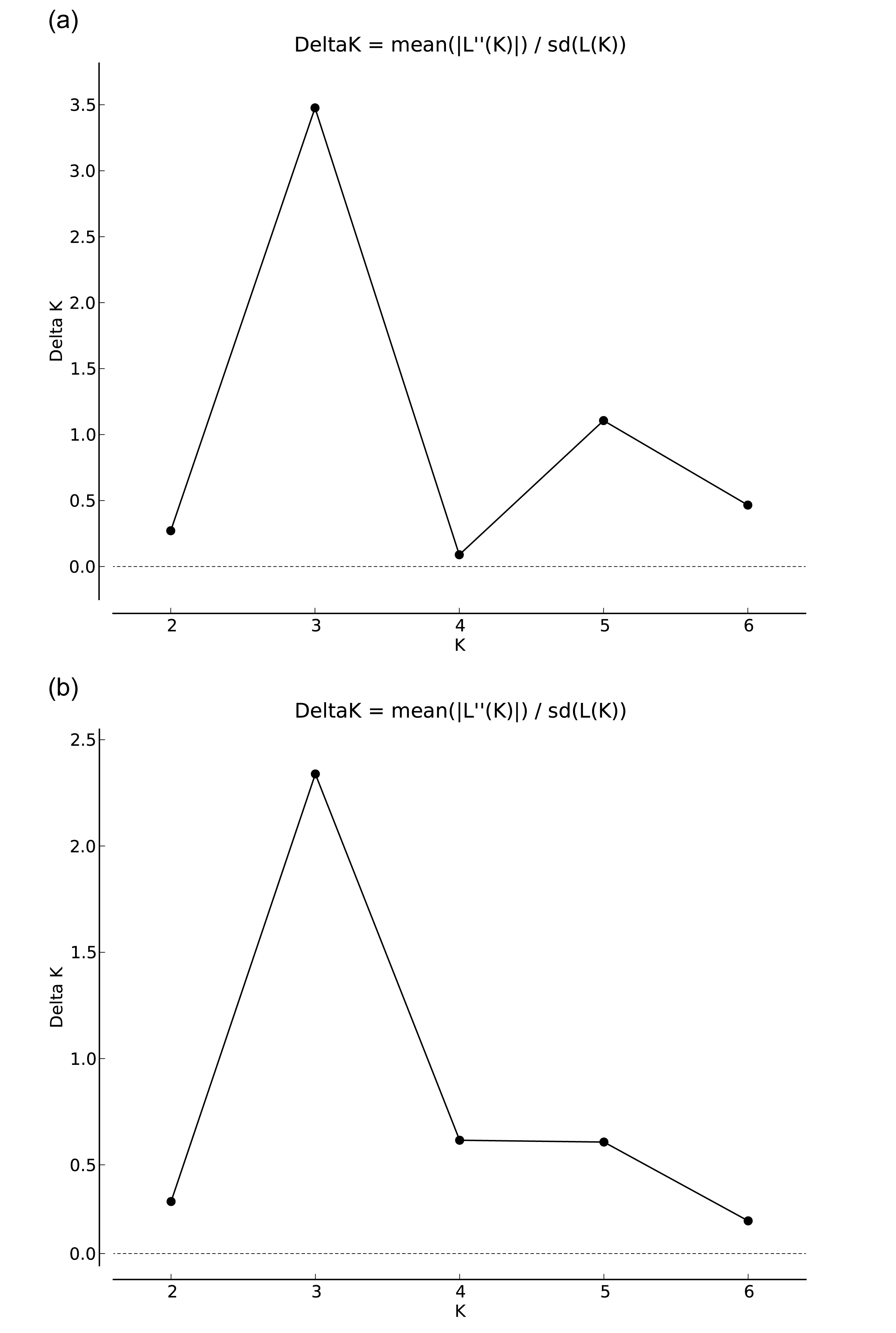

Supplement: Supplementary file 1 [file cells-08-00377-s001.zip › cells-420779-supplementary/suppl-proof/Figure S5 Delta K.tif]

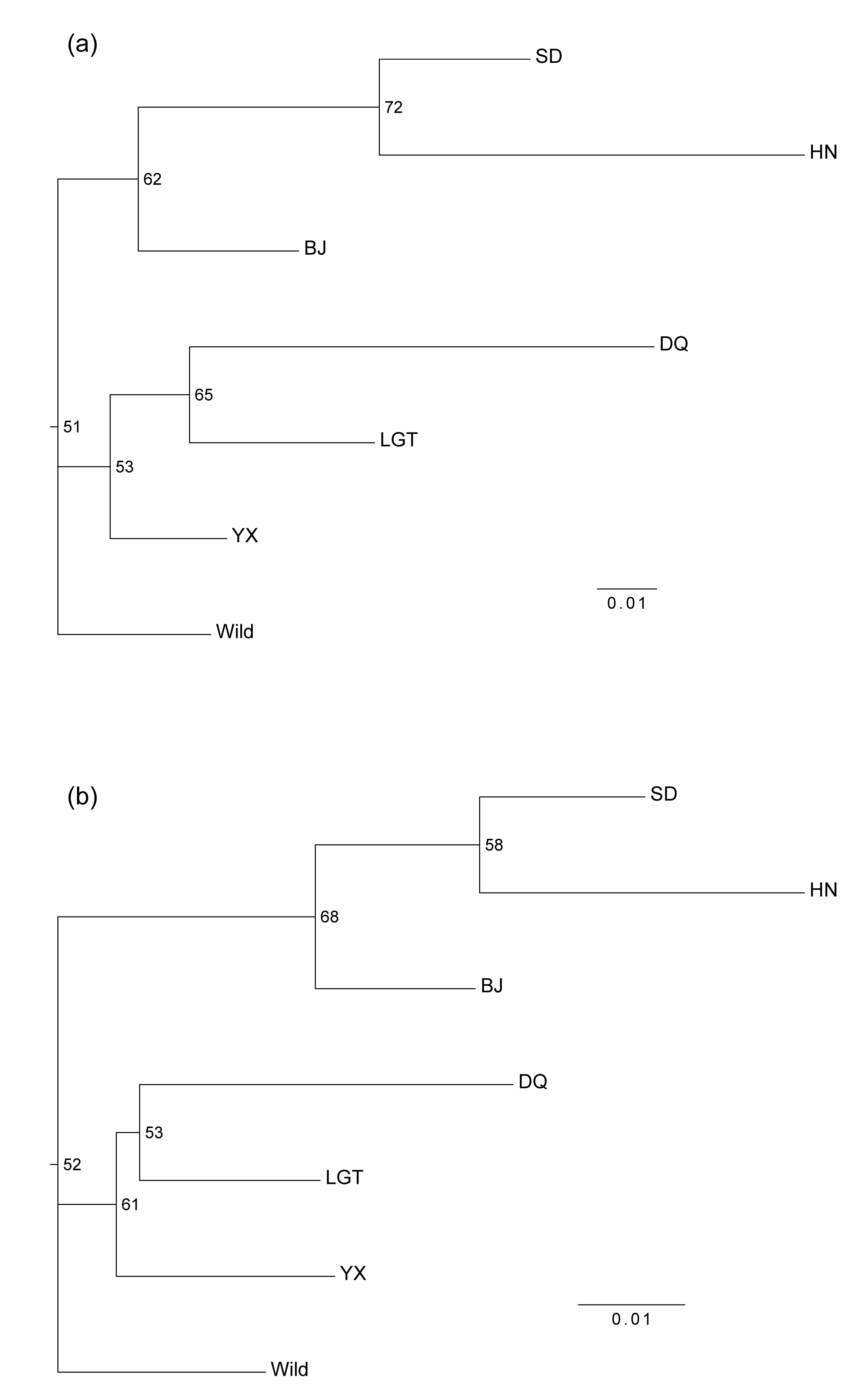

Supplement: Supplementary file 1 [file cells-08-00377-s001.zip › cells-420779-supplementary/suppl-proof/Figure S6 Population phylogenetic tree.tif]
